# Cone photoreceptor phosphodiesterase *PDE6H* inhibition regulates cancer cell growth and metabolism, replicating the dark retina response

**DOI:** 10.1186/s40170-023-00326-y

**Published:** 2024-02-13

**Authors:** Ceren Yalaz, Esther Bridges, Nasullah K. Alham, Christos E. Zois, Jianzhou Chen, Karim Bensaad, Ana Miar, Elisabete Pires, Ruth J. Muschel, James S. O. McCullagh, Adrian L. Harris

**Affiliations:** 1 Molecular Oncology Laboratories, Department of Medical Oncology, John Radcliffe Hospital, Weatherall Institute of Molecular Medicine, University of Oxford, Oxford, OX3 9DS UK; 2 Department of Engineering Science, Institute of Biomedical Engineering (IBME), University of Oxford, Old Road Campus Research Building, Oxford, OX3 7DQ UK; 3 Department of Oncology, University of Oxford, Old Road Campus Research Building, Oxford, OX3 7DQ UK; 4 Department of Chemistry, University of Oxford, Mansfield Road, Oxford, OX1 3TA UK

## Abstract

**Background:**

*PDE6H* encodes PDE6γ′, the inhibitory subunit of the cGMP-specific phosphodiesterase 6 in cone photoreceptors. Inhibition of PDE6, which has been widely studied for its role in light transduction, increases cGMP levels. The purpose of this study is to characterise the role of *PDE6H* in cancer cell growth.

**Methods:**

From an siRNA screen for 487 genes involved in metabolism, *PDE6H* was identified as a controller of cell cycle progression in HCT116 cells. Role of *PDE6H* in cancer cell growth and metabolism was studied through the effects of its depletion on levels of cell cycle controllers, mTOR effectors, metabolite levels, and metabolic energy assays. Effect of *PDE6H* deletion on tumour growth was also studied in a xenograft model.

**Results:**

*PDE6H* knockout resulted in an increase of intracellular cGMP levels, as well as changes to the levels of nucleotides and key energy metabolism intermediates. *PDE6H* knockdown induced G1 cell cycle arrest and cell death and reduced mTORC1 signalling in cancer cell lines. Both knockdown and knockout of *PDE6H* resulted in the suppression of mitochondrial function. HCT116 xenografts revealed that *PDE6H* deletion, as well as treatment with the PDE5/6 inhibitor sildenafil, slowed down tumour growth and improved survival, while sildenafil treatment did not have an additive effect on slowing the growth of PDE6γ′-deficient tumours.

**Conclusions:**

Our results indicate that the changes in cGMP and purine pools, as well as mitochondrial function which is observed upon PDE6γ′ depletion, are independent of the PKG pathway. We show that in HCT116, *PDE6H* deletion replicates many effects of the dark retina response and identify *PDE6H* as a new target in preventing cancer cell proliferation and tumour growth.

**Supplementary Information:**

The online version contains supplementary material available at 10.1186/s40170-023-00326-y.

## Background

Metabolic pathways regulate cell cycle progression by contributing to biosynthesis [1–3] in a sustainable manner, limiting reactive oxygen species (ROS) exposure of newly synthesised DNA [4, 5], and providing the cell with ATP at critical points [6, 7]. However, which metabolic genes regulate G1S and G2M in particular are still not fully elucidated. We therefore conducted an siRNA screen of 487 genes involved in energy metabolism or associated with an in vivo hypoxia gene signature [8] in the HCT116 colorectal cancer cell line. As a result of our screen, we found the cone photoreceptor phosphodiesterase 6H (*PDE6H*) to be a regulator of cell cycle progression, as a mediator of nucleotide synthesis and energy metabolism.

PDE6 is a cGMP-specific phosphodiesterase that hydrolyses cGMP to GMP and the only member of the PDE family that has an inhibitory subunit. This inhibitory unit PDE6γ is encoded by *PDE6G* in rod, and PDE6γ′ is encoded by *PDE6H* in cone cells. Besides its inhibitory role in the PDE6 complex, PDE6γ′ acts as a chaperone and is necessary for PDE6 complex function [9].

Light signal transduction starts as light from the enviroment hits the retina, resulting in the activation of the PDE6 complex through the release of the inhibitory γ-subunits from the catalytic subunits [10]. Activation of the PDE6 complex causes a rapid decrease in cGMP concentration, which leads to the closure of cyclic nucleotide-gated channels in the plasma membrane [11]. When PDE6 is inactive, the cGMP bound to CNG channels sustain a “dark current”, caused by the entry of Na^+^ and Ca^2+^ ions into the photoreceptor cell, depolarising the outer membrane of the retina [10].

cGMP levels, and therefore the PDE6 activity, are diffusion controlled [12]. Subcellular cGMP pools are governed by NO synthases, PDEs, VASP, protein kinases, and cyclic nucleotide-gated and ATP-coupled channels [13–15]. Compartmentalisation and the highly dynamic nature of cGMP signalling pose a challenge to the study of downstream effectors of cGMP.

*PDE6H* has been observed as part of a gene signature related to poor prognosis in non-small lung cancer [16]. *PDE6H* mRNA is also overexpressed in renal carcinoma (chromophobe renal cell and renal pelvis urothelial carcinomas) [17], prostate carcinoma [18], and breast (ductal in situ) [19] cancer cells, compared to normal tissue.

However, since the mechanistic effects of *PDE6H* in non-retinal cells are poorly understood and because of its identification via the screen, we investigated the pathways it acts through in cancer cell lines. Previous work has shown that pharmacological inhibition of other cGMP-specific PDEs, PDE5, and PDE9 results in antiproliferative effects in cancer cells and alters mitochondrial function in fat cells, respectively. While the effect of PDE6 on cell growth and metabolism has not been studied beyond its retinal function [20], there are clear differences in nucleotide pools and basal metabolism of dark vs light retina. The effect of inhibition cGMP-specific PDE on cellular metabolism and the G1 arrest inducing results of *PDE6H* knockdown in HCT116 brought to our attention *PDE6H* as a controller of cancer cell proliferation and metabolism.

## Results

### siRNA screen identified metabolic genes associated with G1/S and G2/M regulation

**Fig. 1:**
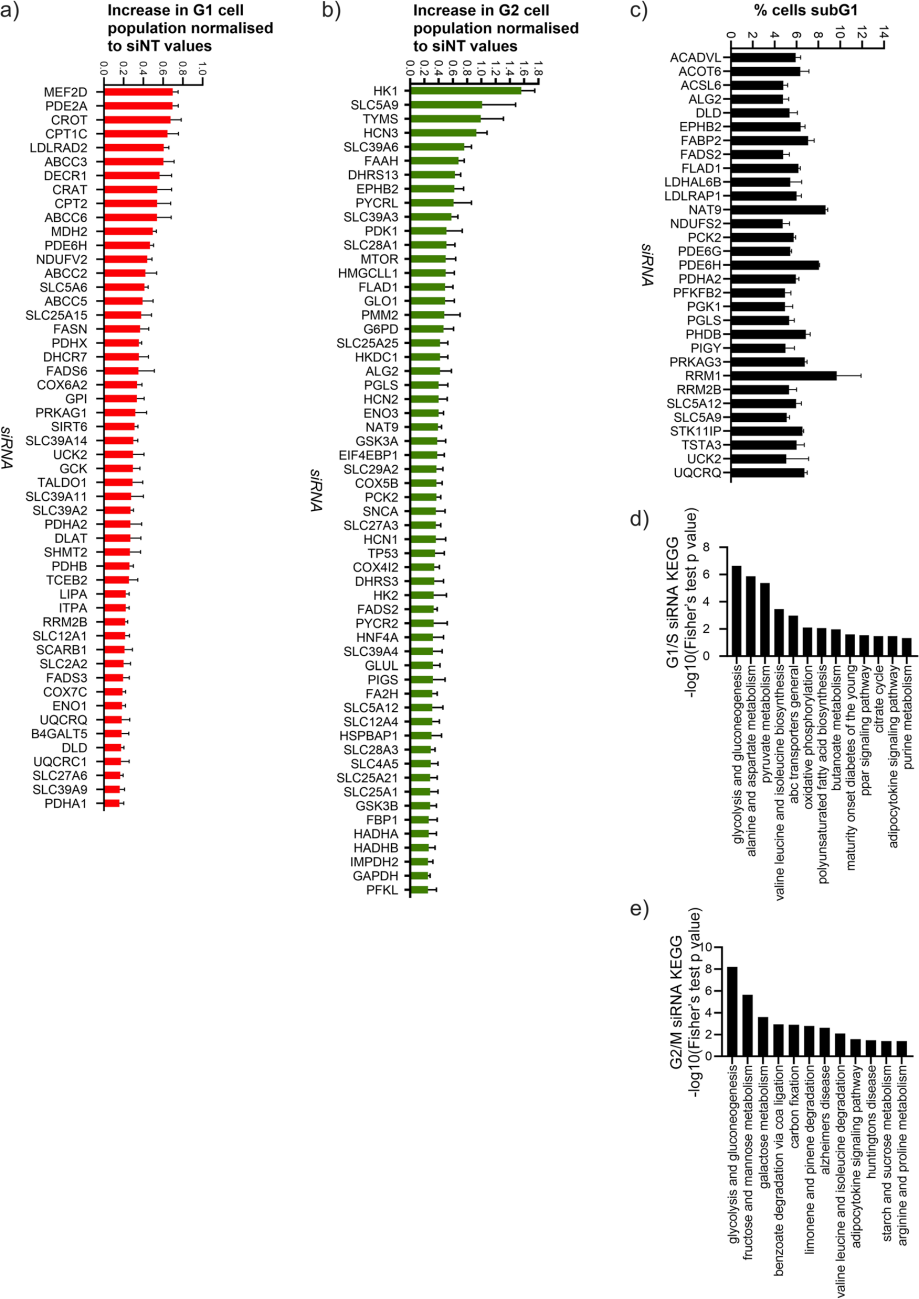
Knockdown of metabolic genes caused G1/S or G2/M arrest in HCT116 cells. **a** G1/S arrest and **b** G2/M arrest inducing siRNAs, which caused an increase in G1 population of > 0.15 or G2 population of > 0.25. The mean of normalised differences in G1 or G2 populations were greater than two standard deviations for the siRNAs presented. Hits shown have caused an unnormalized increase of > 5% in G1 or G2 populations, in at least two independent screens where *n* = 3. **c** Percentage of cells with sub-G1 DNA content were presented as an estimate of cell death. Genes, the knockdowns of which caused a sub-G1 population greater than 4%, are presented. The means are shown; error bars represent SD (*n* = 3). **d** KEGG pathway classification of G1/S targets and of **e** G2/M targets

A custom siRNA screen using HCT116 cells was conducted consisting of 487 metabolic genes (Table S1) encoding regulatory enzymes of cell metabolism, metabolite transporters, and isozymes of genes that were previously shown to be amplified in at least three types of primary tumours [8]. The results of the screen were evaluated in two groups: G1/S (Fig. 1a) and G2/M (Fig. 1b) arrest-inducing siRNAs.

#### Effects of transfection with siRNAs on the G1/S and G2/M populations

Knockdown of genes involved in mitochondrial metabolism, genes that encode enzymes which provide substrates for the TCA cycle, including pyruvate dehydrogenase (PDH) complex components PDHA1, PDHA2, PDHB, PDHX, MDH2, and DLD and fatty acid oxidation (FAO) enzymes CRAT, CROT, CPT1C, CPT2, and DLAT caused an G1/S arrest. Similarly, knockdown of genes coding for enzymes mediating fatty acid desaturation and synthesis, FASN, SREBF2, FADS3, FADS6, DHCR7, LIPA, LDLRAD2, and SCARB1, resulted in G1/S arrest (Fig. 1a).

Similar to previous reports that show an increased FAO in quiescent cells, which are more vulnerable to inhibition of this pathway than proliferating cells [21], we observed that the knockdown of several fatty acid oxidation (CROT, CRAT, CPT1C, CPT2, DECR1, PDHA, PDHX, DLAT) and polyunsaturated fatty acid synthesis genes (FADS6, FADS3) led to G1/S arrest (Fig. 1a).

In addition to the inhibition of the PDH complex genes, knockdown of other enzymes with NAD^+^ as their cofactor, including *SIRT6*, *MDH2*, *DLD*, and electron transport chain components *NDUFV2 COX6A2*, *COX7C*, *UQCRQ*, and *UQCRC1* all caused arrest in G1/S.

Knockdown of other mitochondria-related genes including *GLUL*; beta-oxidation genes *HADHA*, *HADHB*, and *HADH*; and electron transport chain genes *COX5B*, *COX4I2*, and *COX7B2* resulted in G2/M arrest.

Amongst the G2/M arrest-inducing siRNAs were those that silenced genes involved in glycolysis and gluconeogenesis such as *HK1*, *HK2*, *HKDC1*, *G6PD*, *G6PC2*, *FBP1*, *PGLS*, and *PFKL* (Fig. 1e). Most organelle remodelling occurs during G2/M, including the plasma membrane, nuclear membrane, endoplasmic reticulum (ER), the Golgi, and mitochondria. Gluconeogenesis becomes crucial for growth at this stage [22, 23]. G6P is a membrane-bound enzyme of the ER, the site for protein glycosylation, which *ALG2*-encoded mannosyltransferase partakes in.

Protein glycosylation is initiated by hexokinases, which we also identified as G2/M targets [24, 25]. ER resident oxidoreductases *DHRS3* and *DHRS13* were other G2/M targets. P53 target DHRS3 plays a role in the accumulation of lipid droplets [26] which are produced in ER during G2 [27] and are then dispersed during S [28]. NADPH-producing *PYCRL* and *GLO1*, which are important for ROS detoxification, were amongst G2/M targets.

siMEF2D transfected HCT116 cells were arrested in G1/S, similar to the G1 arrest observed upon MEF2D inhibition in gallbladder carcinoma cell lines [29]. Knockdown of genes involved in DNA repair and de novo nucleotide synthesis: *ABCC2*, *ABCC3*, *ABCC5*, *ABCC6*, *SIRT6*, *ITPA*, *TCEB2*, and *RRM2B* also caused G1 arrest. This was expected as the metabolite interconversions facilitated by these enzymes and transporters are necessary for DNA synthesis and crucial for G1/S transition. Amongst such genes that resulted in growth inhibition after knockdown were G1/S targets *LDLRAD2*, *GCK*, and *ITPA*, as well as G2/M targets *mTOR*, *PDK1*, *GSK3A*, and *GSK3B*. *TYMS* knockdown resulted in G2/M arrest which is consistent with the reports of the production of dTMP being higher in S/G2M than in G1 [30].

Our results are supported by previous studies that explored changes in mRNA levels, translation and protein expression of genes in HeLa cells across the cell cycle [31]. Genes encoding PDH and UQCR enzymes, *RRM2*, *NDUFS2*, LDLR chaperone *MESDC2*, and DHCR member *DHCR7*, all have their peak mRNA levels in G1. CPT members have peak mRNA levels in S; these genes are amongst our G1/S targets. *PGLS*, which is a G2/M target, has peak levels of translation in G2 [31].

Knockdown of purine metabolism genes *PDE2A* and *PDE6H* also caused G1 arrest. *PDE2A* is known to regulate p21 activity, and its suppression inhibits formation of cyclin D1-CDK complexes [32] which are necessary for G1/S transition. The relation of *PDE6H*, a significant target of this screen, to cancer was unknown.

#### Effects of transfection with the metabolic gene siRNA library on the sub-G1 population

siRNAs that resulted in an increase of sub-G1 HCT116 population (Fig. 1c) included those that arrested cells in G1/S; ACADVL, DLD, PDE6H, PDHA2, PDHB, PIGY, RRM2B, and UQCRQ; those that arrested cells in G2/M; EPHB2, FADS2, FLAD1, GAPDH, NAT9, PCK2, PGLS, and SLCA9; and those targeting genes that were not identified as cell cycle regulating targets of the screen: ACOT6, ACSL6, ACSS2, FABP2, LDHAL6B, LALRAP1, NDUFS2, PDE6G, PFKFB2, PGK1, PRKAG3, RRM1, SLC5A10, SLC5A12, STK11IP, and TSTA3.

It was previously reported that FAO helps clear toxic metabolic intermediates such as ceramides, and inhibition of FAO also leads to apoptosis [21]. Likewise, our results show increase in sub-G1 population cells upon knockdown of beta-oxidation enzymes, including pyruvate dehydrogenase complex components: ACADVL, DLD, FLAD1, PDHA2, and FADS2 (Fig. 1C).

#### *PDE6H* knockdown induced late G1 arrest and cell death in cancer cells

We determined the effect of knockdown of *PDE6H* on HCT116 cells, a breast cancer cell line (MDA-MB-436), and a non-small cell lung cancer cell line (NCI-H23) all cultured at 5-mM glucose (the physiological concentration). The latter two cell lines have copy number amplifications of *PDE6H* [33].

HCT116, NCI-H23, and MDA-MB-436 have mutations in oncogenes and tumour repressors which govern the G1/S progression. KRAS mutations in HCT116 (G13D) and NHI-H23 (G12C) lead to upregulated RAS/ERK/AKT signalling [34]. Additionally, HCT116 has a mutated *PIK3CA* that results in cyclin D1 upregulation and constitutively active S6K and 4EBP [35]. Both mutations interfere with the G1-S cell cycle arrest [36].

In cancer cells with RB loss such as MDA-MB-436, cell cycle progression is independent of CDK4/6 signalling, and cyclin E drives RB activity [37]. This cell line also has low levels of p21 [38]. Additionally, HCT116 has mutated *CDKN2A*, while NHI-H23 and MDA-MB-436 have *TP53* loss-of-function mutations [39]. Thus, while the final results of *PDE6H* knockdown may indicate a major site of arrest in common, there are differences in which cyclins are regulated.

**Fig. 2:**
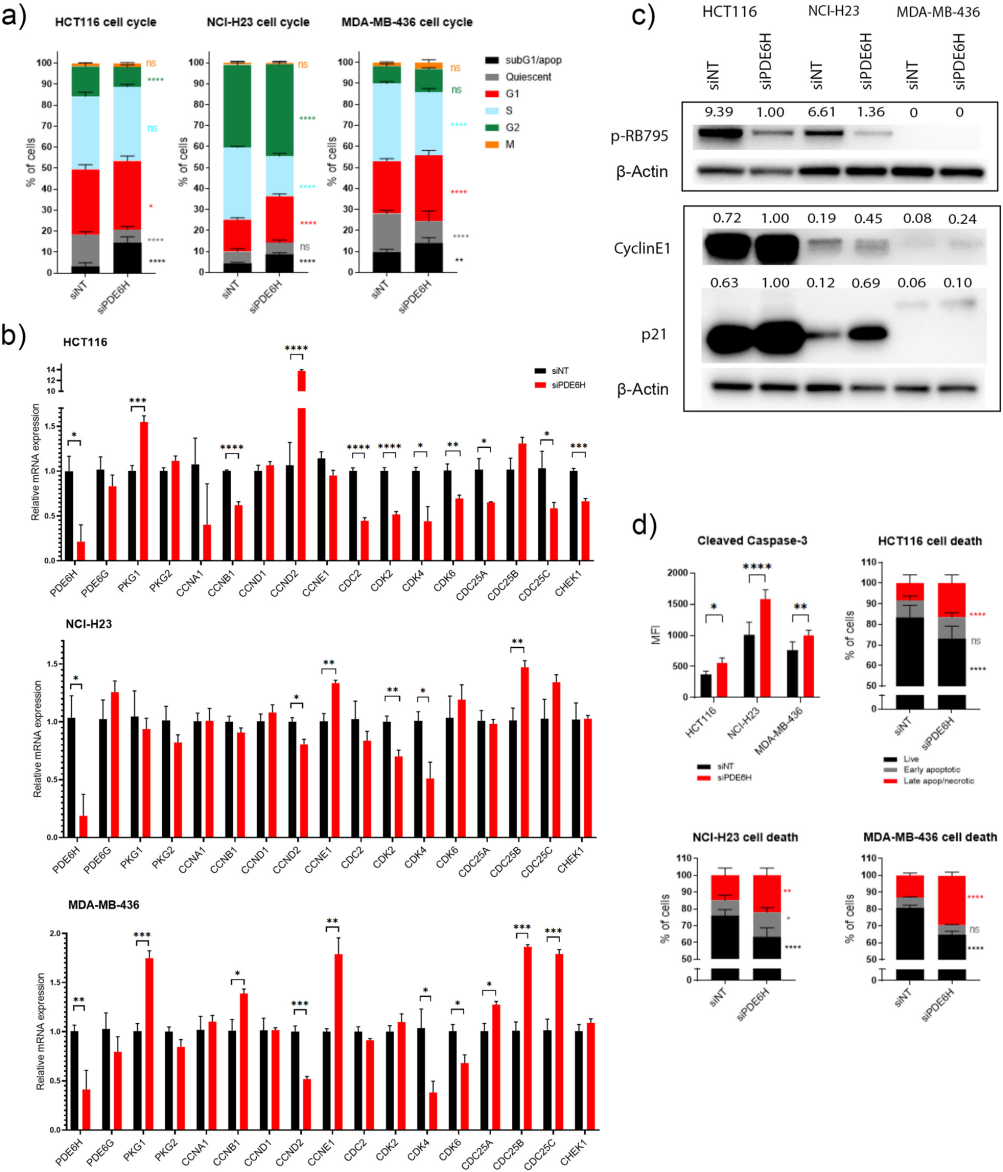
*PDE6H* knockdown induced cell cycle arrest and cell death in HCT116, NCI-H23, and MDA-MB-436. **a** Flow cytometry analysis revealed the percentage of cells in each cell cycle stage relative to the total number of viable cells. *N* = 6 biological replicates. **b** mRNA levels of *PDE6G, PDE6H, PKG* genes, and cell cycle regulators were measured following *PDE6H* knockdown. **c** Immunoblotting revealed that the levels of cell cycle regulators p21 and cyclin E1 increased upon *PDE6H* depletion. P-RB-795 levels decreased in HCT116 and NCI-H23. *N* = 3 biological replicates, quantifications of bands are normalised to those of β-actin values. **d** Cleaved caspase 3 + cells increased upon *PDE6H* knockdown in all three cell lines. *PDE6H* knockdown lowered percentage of live HCT116, NCI-H23, and MDA-MB-436 cells, increasing those of late apoptotic/necrotic cells. *PDE6H* knockdown also increased percentage of early apoptotic NCI-H23 cells. *N* = 6 biological replicates; error bars represent standard deviation (*p < 0.05, **p < 0.01, ***p < 0.001, ****p < 0.0001)

*PDE6H* knockdown resulted in an increase of sub-G1 populations in all three cell lines (Fig. 2a). Similarly, G1 populations increased in all three cell lines. NCI-H23 and MDA-MB-436 had lower percentage of cells in S-phase, but this was not the case for HCT116. Upon *PDE6H* knockdown, the percentage of G2 cells decreased in HCT116 and increased in NCI-H23 compared to control. M-phase populations did not change. Thus, G1 block and increase in sub-G1 populations were the most consistent effects of *PDE6H* knockdown.

*PDE6H* mRNA levels, as expected, were suppressed in the siPDE6H transfected HCT116, NCI-H23, and MDA-MB-436 (Fig. 2b). There was no compensatory increase in *PDE6G* mRNA expression. While *PKG1* mRNA increased in HCT116 and MDA-MB-436, no change was observed in protein levels (results not shown). RNAseq gene expression of *PDE6H* was confirmed in all three cell lines [40].

*CDC2/CDK2*, *CCNA*, and *CCNB* mRNA levels and their translation peaks are in S, S/G2/M, and G2/M stages, respectively, while the maxima for *CDK4* and *CCNE1* are observed in G1 and G1/S, respectively [31, 41]. The mRNA and translation level maxima of *CDK6*, which plays a role in G1 progression as well as differentiation, were observed in G2 and S, respectively [42, 43]. Our results showed that *CDK4* mRNA levels decreased in all three cells lines upon *PDE6H* knockdown (Fig. 2b). Additionally, mRNA levels of at least of two of genes regulated by p21/RB: *CDC2*, *CDK2*, *CDK4*, *CDK6*, *CDC25A*, and *CHEK1* were lower in all three cell lines upon *PDE6H* knockdown.

There is heterogeneity in the dependence of different cancer cell lines on cell cycle regulators. Cyclin E can bypass the dependence on CDK4/6, cyclin D1 can regulate CDK2 activity, and CDC2 can drive cell cycle progression of cell lines that are resistant to G1/S regulation [37]. Cyclin D-dependent cells express low levels of cyclin E and CDK2A; HCT116 is likely cyclin D1 dependent [44]. On the other hand, NHI-H23 is likely cyclin E1 driven since in the presence of WT *PI3KA* and mutated *KRAS*, cyclin D1 is bypassed. We show that *CCND2* mRNA levels were higher in HCT116 upon *PDE6H* knockdown, which can be attributed to this cell line’s *CDKN2A* mutation. In *TP53*-mutant cell lines NHI-H23 and MDA-MB-436, *CCND2* mRNA levels were lower, and *CCNE2* mRNA levels were higher.

All CDC25s control G2/M progression, while CDC25A also regulates G1/S [45–48]. We showed that *CDC25A* and *CDC25C* mRNA levels were lower in HCT116. mRNA levels of G2M effectors *CCNA1*, *CCNB1*, and *CDC25C* were lower in HCT116 which had a lower G2/M population upon *PDE6H* knockdown (Fig. 2a). On the other hand, NCI-H23 and MDA-MB-436, which have higher G2/M populations upon *PDE6H* knockdown, in addition to the G1 arrest, had higher mRNA levels of at least two CDC25 genes (Fig. 2b).

Cyclin E1 protein levels were higher in all three cell lines, as well as *CCNE1* mRNA levels in NCI-H23 and MDA-MB-436 (Fig. 2c). There was also a marked increase in p21 levels and decrease in p-RB protein levels in HCT116 and NHI-23 (Fig. 2c). Taken together, our results indicate a late-G1 arrest for HCT116 and NCI-H23, similar to a profile of cell cycle arrest induced by rapamycin [49]. The *TP53*-mutant and RB-deficient cell line MDA-MB-436 had higher levels of *CCNB1* mRNA upon *PDE6H* knockdown.

*PDE6H* depletion resulted in the downregulation of G1/S regulating CDKs and cyclins and an increase in p21 (Fig. 2b and c). P21 is required for p53-induced G1 arrest due to DNA damage [50] and has been shown to result in G1 arrest in HCT116 as a result of metabolic dysregulation in the absence of DNA damage [51, 52]. *PDE6H* depletion resulted in G1 arrest and cell death irrespective of the p53 status (Fig. 2d).

*PDE6H* knockdown also caused cell death, increasing the percentage of late apoptotic/necrotic cells as well as cleaved caspase 3+ levels in HCT116, MDA-MB-436, and NCI-H23 (Fig. 2d). Early apoptotic cell populations also increased in NCI-H23.

### Depletion of the inhibitory PDE6 subunit PDE6H decreases phosphodiesterase activity and regulates mTORC1 activation

**Fig. 3:**
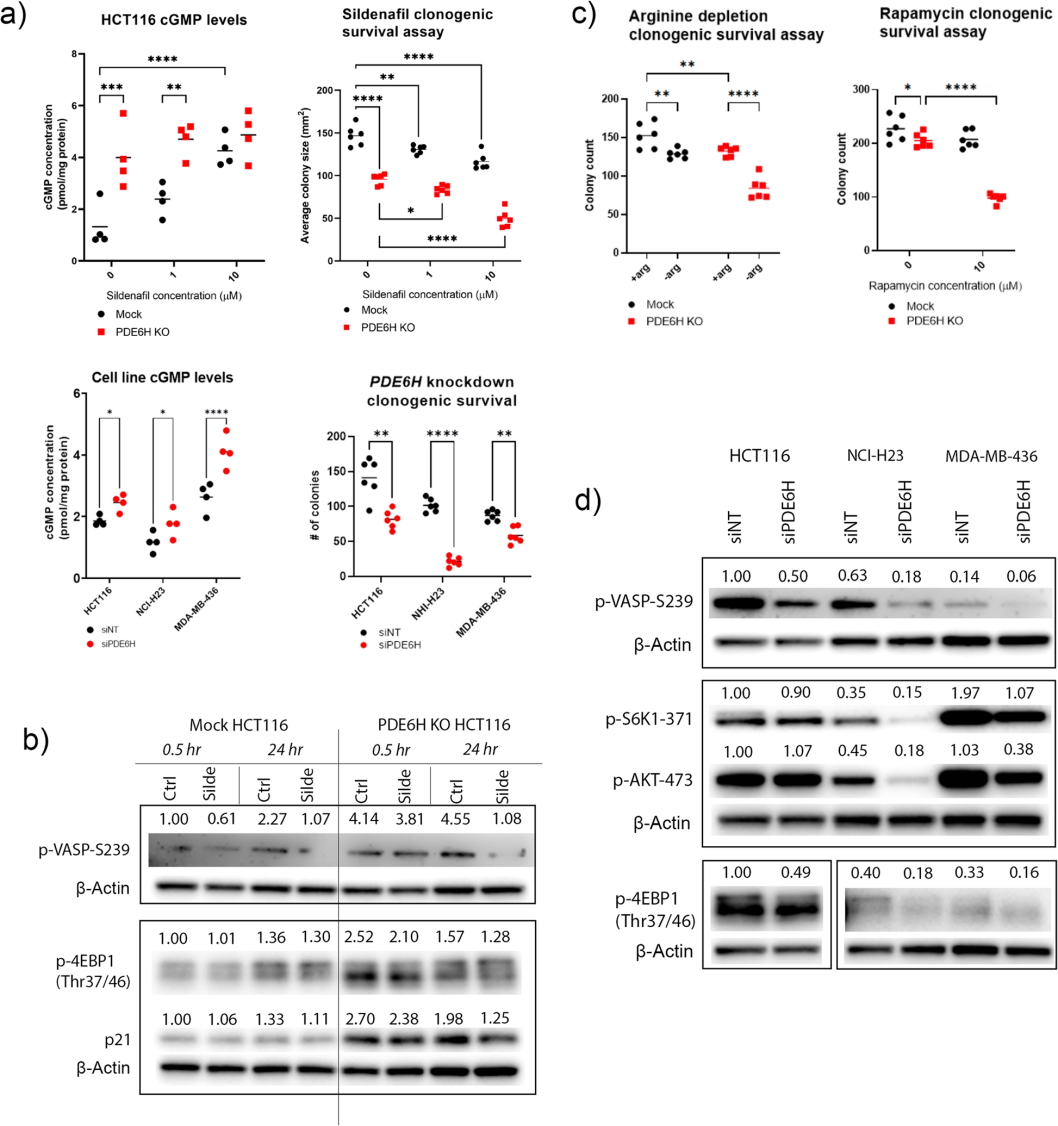
*PDE6H* depletion increased intracellular cGMP levels and lowered mTORC1 activity. **a** cGMP levels of *PDE6H* KO HCT116 compared to mock control following treatment with 10-μM sildenafil citrate for 30 min (*n* = 4), colony sizes of *PDE6H* KO and mock *PDE6H* after a 10-day incubation with or without sildenafil (*n* = 6), and cGMP levels (*n* = 4) and colony counts of HCT116, NCI-H23, and MDA-MB-436 cells following *PDE6H* knockdown (*n* = 6) were measured. **b** Immunoblot analysis of WT and *PDE6H* KO HCT116 following sildenafil treatment. **c** Clonogenic survivals of *PDE6H* KO and mock HCT116 cells were assessed upon arginine depletion and rapamycin treatment, *N* = 6. *p < 0.05, **p < 0.01, ***p < 0.001, ****p < 0.0001. **d** Immunoblot analysis of HCT116, NCI-H23, and MDA-MB-436 upon *PDE6H* knockdown. Representative images of Western blots are presented in Fig. 2b and d, *n* = 3

To analyse the effects PDE6γ′ depletion in vivo without the confounding effects of siRNA gene silencing, we generated CRISPR-Cas9 *PDE6H* knockout (KO) cells (Figure S1a). *PDE6H* KO HCT116 had higher basal cGMP levels compared to mock control HCT116 (Fig. 3a), indicating lower levels of PDE6 activity in *PDE6H* KO HCT116. Likewise, *PDE6H* knockdown of HCT116, NCI-H23, and MDA-MB-436 cells resulted in elevated cGMP levels and lower clonogenic survival (Fig. 3a). Following a 10-day sildenafil treatment, cGMP levels of mock HCT116 slightly but significantly increased upon sildenafil treatment, still remaining below the levels of *PDE6H* KO cells (Figure S2). cGMP levels of KO cells were not affected by the 10-day sildenafil treatment.

Since *PDE6H* encodes the inhibitory PDE6 subunit, its inhibition was expected to increase PDE6 activity. PDE6 inhibition due to *PDE6H* depletion seemed paradoxical. However, PDE6γ′ has a chaperone function for assembly of an active PDE6 complex [9] as well as enhancing the cGMP binding to PDE6 GAF domain [53]. The chaperone role may therefore predominate. The retina of the Pde6g KO mouse model displays lower levels of PDE activity and higher levels of cGMP compared to WT, both in dark- and light-adapted conditions [54], also supporting the lack of hydrolytic activity of PDE6γ-depleted PDE6 complex. The inhibitory subunits differ structurally, while rod PDE6γ and cone PDE6γ′ can both enhance the stability of rod PDE6 catalytic dimer; cone PDE6 has a greater affinity for PDE6γ′ than for PDE6γ [55].

Next, we attempted to understand if PDE6γ′ inhibition via knockout and knockdown would have similar effects to PDE5/6 inhibition by PDE inhibitor sildenafil [56]. The PDE6γ-binding site of the rod PDE6 overlaps with the PDE5/6 inhibitor sildenafil-binding site. Therefore, while PDE5 does not have an inhibitory subunit, the drug might be allosterically competing with the gamma subunit of PDE6 [57].

Despite the presence of many PDEs and hyperpolarisation-activated, cyclic nucleotide-gated channel (HCN) family members that control the intracellular cGMP levels via breakdown or transport, treatment of *PDE6H* KO HCT116 with the PDE5/6 inhibitor sildenafil did not further increase cGMP levels in contrast to control HCT116 (Fig. 3a). Thus, it is likely that the modulation of PDE6γ activity is sufficient to alter intracellular cGMP levels. At the same time, although untreated *PDE6H* KO HCT116 cells grew into smaller colonies than mock control, sildenafil treatment further decreased colony size (Fig. 3a). This suggested that some residual PDE5 activity was important for colony growth in *PDE6H* KO HCT116.

Immunoblot analysis revealed that p21 levels and levels of mTORC1 pathway downstream effectors changed following *PDE6H* depletion or sildenafil treatment. P-4EBP1 and p21 levels were higher (Fig. 3b) and p-ERK levels lower (data not shown) in *PDE6H* KO HCT116 cells. Sildenafil treatment lowered p-VASP-239 in both mock and KO HCT116 but only after a longer treatment in KO cells. A 24-h sildenafil treatment also decreased p21 levels in both mock and KO cells.

Since *PDE6H* knockout altered mTORC1 activity, we assessed how the clonogenic survival of *PDE6H* KO cells was affected by depletion of arginine, for which mTORC1 acts as a sensor [58]. *PDE6H* KO HCT116 was more sensitive to arginine depletion and treatment with mTORC1 inhibitor rapamycin than mock HCT116 (Fig. 3c).

*PDE6H* knockdown in HCT116, NCI-H23, and MDA-MB-436 decreased protein levels of p-VASP and mTORC1 effector p-4EBP1. P-AKT-473 protein levels of NCI-H23 and MDA-MB-436 also decreased upon *PDE6H* knockdown. The cell cycle arrest was accompanied with lower mTORC1 activity, suggesting that the arrest is due to a “nutritional deficiency” [49]. Elevated p-4EBP1 in KO HCT116, despite lower levels of the protein in *PDE6H* knockdown cell lines, suggests a means of survival during chronic *PDE6H* depletion [59].

Interestingly, depletion of *PDE6H* or sildenafil treatment did not increase p-VASP levels at the cGMP/PKG site Ser239 (Fig. 3b and d). P-VASP levels did not change upon *PDE6H* knockout. However, they were lower after sildenafil treatment in both mock and *PDE6H* KO HCT116. P-VASP levels were also lower in all three cell lines upon *PDE6H* knockdown. This suggested that the downstream effects of cGMP increase due to *PDE6H* depletion or sildenafil treatment were independent from PKG/VASP activity. PDE5 can be activated by direct binding of cGMP to its GAF domains or through phosphorylation by PKG1 at Ser92 [60, 61]. Levels of p-PDE5 were higher in *PDE6H* KO compared to mock HCT116 and increased upon sildenafil treatment (Figure S1b), which suggested that elevated levels of cGMP in these cells led to upregulation of basal PDE5 activity. Our results indicated the presence of PDE5 in its activated state in *PDE6H* KO HCT116 and pointed at a limitation to PDE5 inhibition by sildenafil.

Our results are in accordance with a recent study that has shown that a 3-day treatment of HCT116 with 50-µM sildenafil decreased proliferation without causing VASP phosphorylation, which led to the conclusion that the antiproliferative effect of sildenafil treatment is not dependent on cGMP/PKG signalling [62].

Another group pointed at ROS accumulation as the means of sildenafil’s antiproliferative, cell cycle arrest and apoptosis-inducing effect. This effect, observed upon treatment with sildenafil at non-clinically relevant high concentrations (100 µM), was reversed via pretreatment of cells with NAC [63].

### Effects of PDE6H knockout on nucleotide metabolism and energy transduction pathways

**Fig. 4:**
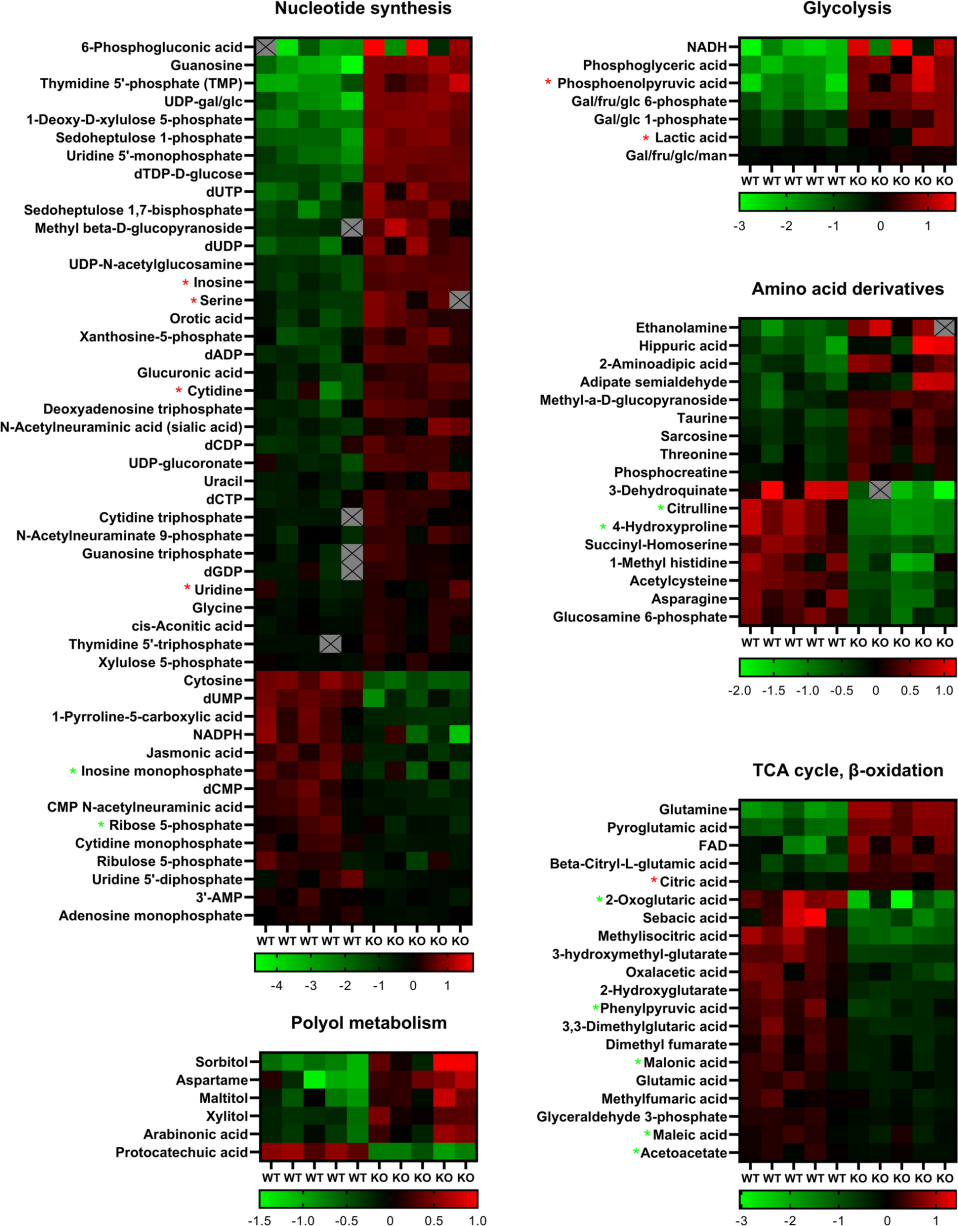
*PDE6H* deletion altered nucleotide abundances as well as intermediates of the TCA cycle and sugar metabolites. There was an accumulation of mostly nucleotide triphosphates and a depletion of monophosphate nucleotides in *PDE6H* KO HCT116 compared with WT HCT116, measured by LC–MS. Red asterisks mark the accumulation, and green shows the depletion of the respective nucleotides upon light transduction [20]. Results are presented in the order of fold change between the KO and WT HCT116. Heatmap represents log_2_ of the fold change of metabolite levels from the average abundances of all samples. Compounds which altered in abundance more than 20% are presented; *N* = 5 tissue culture replicates per experimental group, *Q* < 0.05 (FDR-corrected *p*-value)

As *PDE6H* depletion potentially disrupts cellular purine metabolism by altering GMP homeostasis, and since mTORC1 activity is influenced by purine levels [64], we measured the intracellular levels of nucleotide pools in HCT116 WT control and *PDE6H* KO cells using liquid chromatography-mass spectrometry (LC–MS) [65] (Fig. 4).

*PDE6H* KO HCT116 cells had higher levels of intermediates of glycolysis, pentose phosphate pathway (PPP), and polyol and hexosamine metabolism which supplements glycolysis intermediates, compared to WT HCT116 (Fig. 4). Levels of PPP intermediates 1-deoxy-D-xylulose 5-phosphate, sedoheptulose 1-phosphate, sedoheptulose-1,7-bisphosphate, gal/fru/glc 6-phosphate, gal/glc 1-phosphate, sorbitol, 6-phosphogluconic acid, UDP-glucoronate, XMP, and X5P were higher in *PDE6H* KO HCT116, while those of R5P and ribulose 5-phosphate were lower. Taken together, the results suggest a backlog in ribulose 5-phosphate production through the glucuronic acid pathway and PPP in *PDE6H* KO HCT116.

Important precursors of de novo nucleotide synthesis, glycine, lysine, glutamine, orotate, and GTP were at higher levels in *PDE6H* KO HCT116. Non-phosphorylated and deoxy-di/trinucleotides were generally at higher levels in *PDE6H* KO HCT116. Levels of guanosine, inosine, cytidine, uracil, uridine, TMP, dUTP, dUDP, dADP, dCDP, dATP, dCTP, TTP, CTP, and GTP were higher in *PDE6H* KO HCT116 cells compared to HCT116 WT. AMP, IMP, CMP, dUMP, dCMP, and UDP levels were lower in KO HCT116. Our results indicated a depletion of RNA precursors and an accumulation of DNA precursors. Increased levels of serine and phosphoglyceric acid in *PDE6H* KO HCT116 might be an indicator of upregulation of serine synthesis [66].

#### Comparison of retina dark response with *PDE6H* deletion

Interestingly, certain changes in metabolite levels in *PDE6H* KO HCT116 cells matched those of dark-adapted mice retina (Fig. 4, Figure S3). A study on the effect of phototransduction on nucleotide metabolism showed that the metabolic flux through glycolysis and TCA cycle decreases when light hits the retina [67]. Light, the stimulus for PDE6 activity, causes IMP to accumulate, while most other purine and pyrimidine metabolites are depleted. TCA intermediate 2-oxoglutaric acid is also lower in light-stimulated retina. cGMP, inosine, serine, cytidine, CTP, and uridine are depleted when dark-adapted mice retina is exposed to light. These metabolites were higher in *PDE6H* KO HCT116 cells compared to HCT116 WT; likewise, IMP and R5P were lower in *PDE6H* KO HCT116 cells. PEP and lactic acid were higher in dark retina and *PDE6H* KO HCT116. Similar to Du et al.’s report that light inhibits oxidation of 2-oxoglutarate, levels of this metabolite are higher in the light-exposed retina and WT HCT116, while the opposite trend is observed for citric acid. Several other TCA intermediates were also lower in *PDE6H* KO HCT116 cells, with phenylpyruvic acid, malonic acid, and maleic acid displaying lower levels in *PDE6H* KO versus WT HCT116. Interestingly, this is the same pattern found in the dark retina versus light retina.

Our results also revealed that TCA cycle/OXPHOS intermediates glutamine and FAD, as well as glycolysis intermediates NADH, gal/fru/glc 6-phosphate, gal/glc 1-phosphate, and gal/fru/glc/man, were at higher levels in *PDE6H* KO HCT116. Accumulation of phosphoglycerate and PEP with lower levels of TCA intermediates such as 2-oxoglutaric acid and 2-hydroxyglutarate suggested that there was less conversion into pyruvate, therefore less input from glycolysis to the TCA cycle in *PDE6H* KO HCT116. Our results suggest that 2-oxoglutarate levels remained lower probably due to a lower contribution from reductive glutamine metabolism in *PDE6H* KO HCT116, considering that the glutamine levels of *PDE6H* KO were higher. 2-oxoglutarate/citrate levels were lower in *PDE6H* KO HCT116, which suggested that the TCA cycle may have been active in the reductive direction [68].

#### *PDE6H* knockdown represses mitochondrial function

**Fig. 5:**
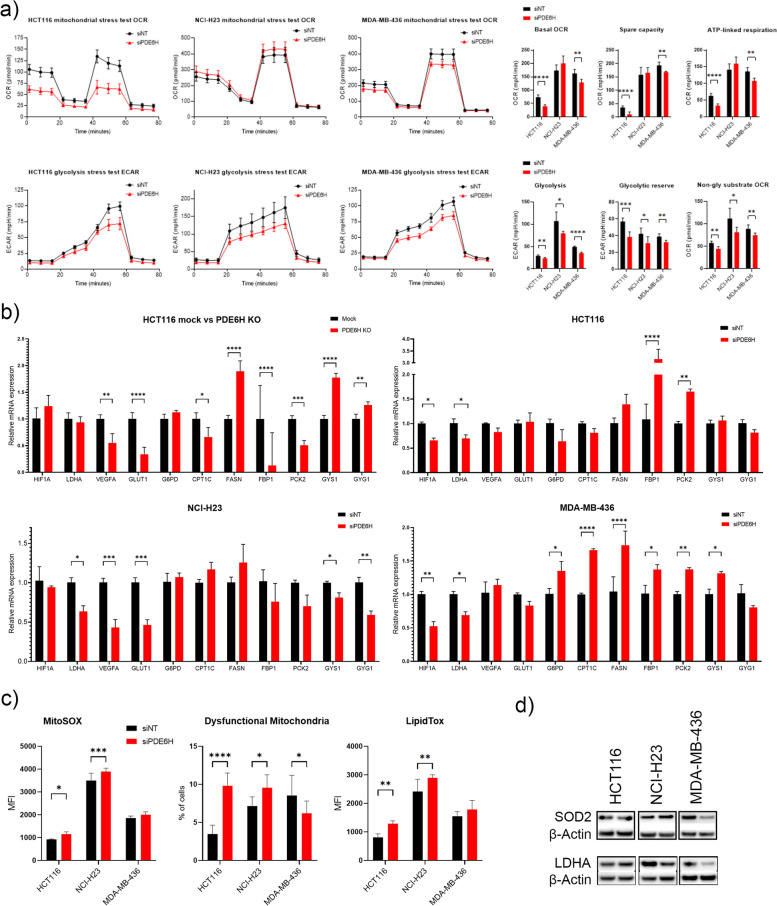
*PDE6H* depletion altered mitochondrial metabolism and glycolysis. **a** *PDE6H* knockdown lowered levels of OXPHOS in HCT116 and MDA-MB-436 and of glycolysis in HCT116, NCI-H23, and MDA-MB-436, *n* = 5. **b** mRNA levels of hypoxia markers and metabolic enzymes were measured upon *PDE6H* depletion, *n* = 4. **c** Neutral lipid droplet levels, as well as percentage of dysfunctional mitochondria and mitochondrial ROS, increased in HCT116 and NCI-H23, whereas percentage of dysfunctional mitochondria decreased in MDA-MB-436. Levels of lipid droplets were indicated by LipidTOX Deep Red and mitochondria ROS by MitoSOX. Percentage of dysfunctional mitochondria was determined as the population of cells with high MitoTracker Green and low MitoTracker DeepRed signal, *N* = 6. *p < 0.05, **p < 0.01, ***p < 0.001, ****p < 0.0001

Having observed the effects of *PDE6H* depletion on glycolysis and TCA cycle intermediates (Fig. 4), we conducted mitochondrial and glycolysis stress tests using the Seahorse XF Analyzer to determine the effects of *PDE6H* knockdown on the oxidative phosphorylation and glycolysis levels of HCT116, NCI-H23, and MDA-MB-436 cell lines (Fig. 5a). Levels of basal OCR, spare respiratory capacity, and ATP-linked respiration were lower in siPDE6H transfected HCT116 and MDA-MB-436. Levels of glycolysis and glycolytic reserve decreased in all three cell lines upon *PDE6H* knockdown. OCR values following glycolytic inhibitor 2-DG injection (data not shown) were used to determine levels of OCR that depend on endogenous non-glycolytic substrates [69]. Non-glycolytic substrate OCR levels were lower in all three cell lines following *PDE6H* knockdown, indicating an overall reduction from possible substrates such as nucleosides, glutamate, fatty acids, or TCA intermediates.

The effects of *PDE6H* knockout and knockdown on the mRNA levels of several metabolic genes were measured (Fig. 5b). These genes included those involved in response to hypoxia, *HIF1A*, *LDHA*, *VEGFA*, *GLUT1*, and *GYS1*, as well as beta-oxidation and fatty acid synthesis genes, *CPT1C* and *FASN*, which belong to the list of G1/S targets (Fig. 1a). mRNA levels of G2/M targets, including the gluconeogenesis rate-limiting enzymes *FBP1* and *PCK2*, and PPP rate-limiting enzyme *G6PD* were also measured.

While there was heterogeneity in the hypoxia response and specific genes varied from cell line to cell line, mRNA levels of hypoxia response genes decreased upon *PDE6H* depletion. *VEGFA* and *GLUT1* mRNA levels were lower in *PDE6H* KO HCT116 compared to WT. *LHDA* mRNA levels decreased in HCT116, NCI-H23, and MDA-MB-436 upon *PDE6H* knockdown. mRNA levels of *HIF1A* decreased in HCT116 and MDA-MB-436, while those of VEGFA decreased in NCI-H23 upon *PDE6H* knockdown.

Trends of changes of mRNA levels of fatty acid, gluconeogenesis, and glycogen metabolism genes varied across cell lines. *FASN* mRNA levels were higher and *CPT1C* lower in *PDE6H* KO HCT116, while both were higher in MDA-MB-436 upon *PDE6H* knockdown. *FBP1* and *PCK2* mRNA levels were lower in *PDE6H* KO HCT116, while they were higher in HCT116 and MDA-MB-436 upon *PDE6H* knockdown. *GYS1* and *GYG1* mRNA increased in *PDE6H* KO HCT116, while they decreased in *PDE6H* knockdown in NCI-H23 cells.

*FBP1* and *PCK2* mRNA levels were lower in *PDE6H* KO compared to WT HCT116. They increased in HCT116 and MDA-MB-436 upon *PDE6H* knockdown. *GYS1* and *GYG1* mRNA levels were higher in *PDE6H* KO HCT116. These mRNAs were lower in NCI-H23, while *GYS1* mRNA was higher in MDA-MB-436 upon *PDE6H* knockdown.

The increase in *CPT1C* mRNA expression in MDA-MB-436 might indicate an increase in TCA cycle input from beta oxidation, leading to a smaller decrease of OXPHOS in MDA-MB-436 than in HCT116 upon *PDE6H* knockdown. Results indicated an increase in mRNA levels of PPP genes in MDA-MB-436.

There was an increase in lipid droplets, measured by LipidTOX signal in HCT116 and NCI-H23 cells (Fig. 5c). Percentage of dysfunctional mitochondria and MitoSOX signal, which is an indication of mitochondrial ROS, both increased in HCT116 and NCI-H23 cells. Levels of mitochondrial antioxidant protein SOD2, as well as those of the aerobic glycolysis enzyme LDHA, decreased in MDA-MB-436 upon *PDE6H* knockdown (Fig. 5d). LDHA decreased in NCI-H23, while SOD2 levels remained the same; neither marker changed in HCT116 upon *PDE6H* knockdown. Increase in mRNA levels of antioxidant enzyme G6PD in MDA-MB-436 might help alleviate the effect of lower levels of SOD2 [70].

### PDE5/6 inhibitor sildenafil compared to *PDE6H* deletion

**Fig. 6:**
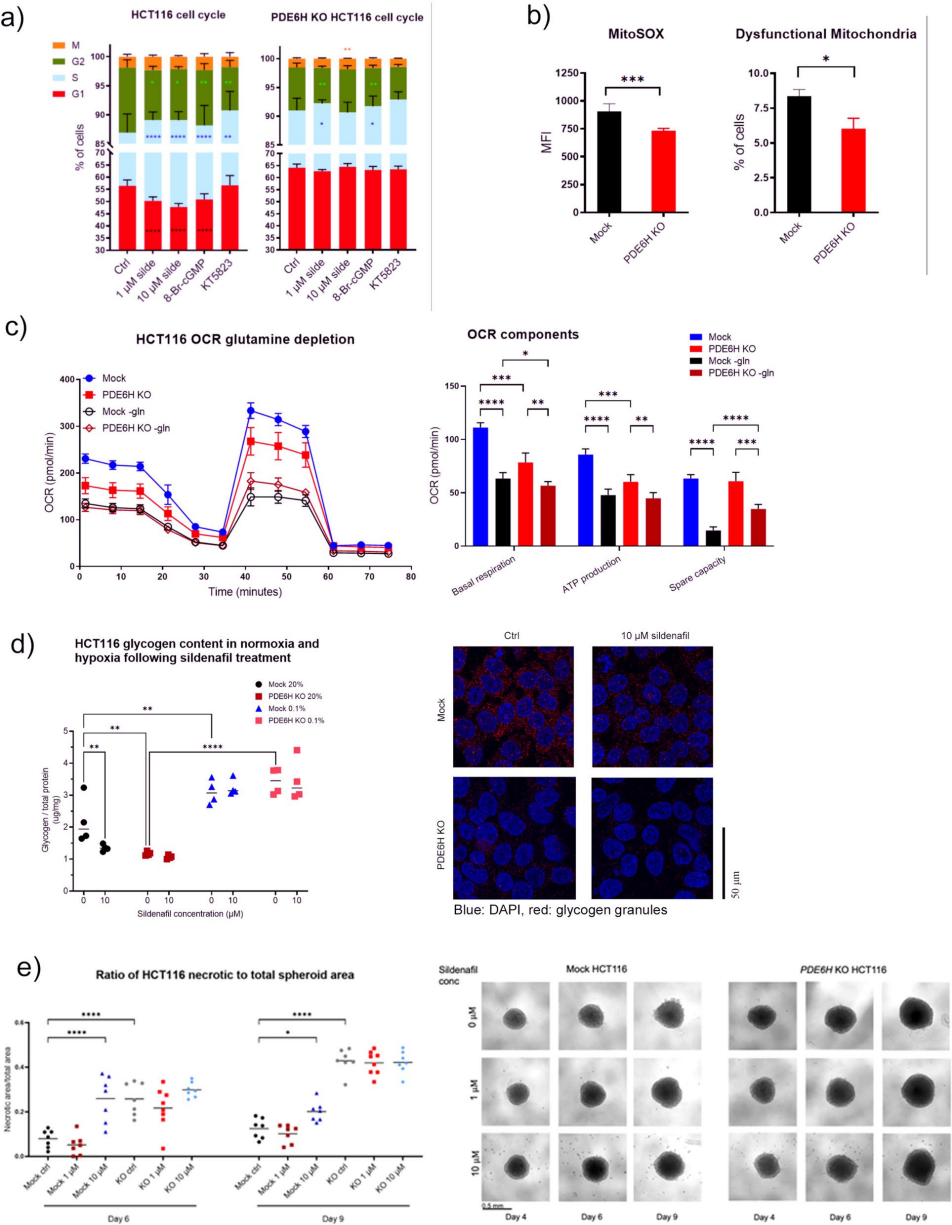
Effects of sildenafil treatment and *PDE6H* KO on cell cycle arrest, glycogen levels, and spheroid necrosis in HCT116 cells. **a** Cell cycle distribution of mock and *PDE6H* KO HCT116 cells following treatment with sildenafil, cGMP analogue 8-Br-cGMP (10 μM), and PKG inhibitor KT5823 (1 μM). **b** Levels of mitochondrial ROS and percentage of dysfunctional mitochondria in mock and *PDE6H* KO HCT116 cells, *n* = 5. Median MitoTOX signal fluorescence intensity indicated mitochondrial ROS levels. Percentage dysfunctional mitochondria were denoted as the fraction of live cells with high MitoTracker Green and low MitoTracker DeepRed signal.* c* Seahorse mitochondrial test was conducted after 20-h glutamine depletion. **d **Glycogen enzymatic assay results and glycogen granule immunofluorescent staining of mock and *PDE6H* KO HCT116 following sildenafil treatment, in normoxia and hypoxia. Red, glycogen granules; blue, DAPI. **e** *PDE6H* depletion effects on the necrotic/viable HCT116 spheroid volume ratio, *N* = 10. *p < 0.05, **p < 0.01, ***p < 0.001, ****p < 0.0001

Sildenafil inhibits both PDE5 and PDE6; therefore, to assess if there was compensation for PDE6 loss by PDE5, effects of sildenafil treatment were investigated. Sildenafil (1 µM and 10 µM) treatment induced S-phase cell cycle arrest in both mock and *PDE6H* KO (1 µM) HCT116, while cGMP analogue 8-Br-cGMP increased S population only in mock HCT116 (Fig. 6a). *PDE6H* KO HCT116 had lower levels of mitochondrial ROS and higher levels of dysfunctional mitochondria than mock HCT116 (Fig. 6b).

The Seahorse mitochondrial stress test was conducted following a 20-h glutamine depletion (Fig. 6c). *PDE6H* KO HCT116 had lower basal and ATP-linked OXPHOS than mock HCT116. Glutamine depletion lowered basal, ATP-linked respiration and spare capacity to a greater extent in mock HCT116, than in *PDE6H* KO HCT116. Spare capacity of glutamine-depleted *PDE6H* KO HCT116 was higher than that of glutamine-depleted mock HCT116. This suggests an adaptation in *PDE6H* KO cells to use other TCA cycle substrates, such as pyruvate or fatty acids.

Both *PDE6H* knockout and sildenafil treatment lowered HCT116 glycogen levels (Fig. 6d). As expected, glycogen levels were higher under hypoxia [71] in both mock and *PDE6H* KO at similar maximum levels. The increase in glycogen under hypoxia in either cell line was not affected by sildenafil treatment. Immunofluorescence (IF) staining also showed glycogen depletion in mock HCT116 upon sildenafil treatment and lower levels of glycogen in *PDE6H* KO HCT116 compared to mock. Under normoxia, sildenafil treatment lowered glycogen content of mock HCT116 to similar levels as *PDE6H* KO HCT116, without affecting the levels of the latter.

The necrotic/total spheroid area ratio was higher in *PDE6H* KO compared to mock HCT116 (Fig. 6e). A total of 10-μM sildenafil increased this ratio in mock HCT116, but not in *PDE6H* KO HCT116. Since *PDE6H* depletion inhibited proliferation as well as disrupting spheroid growth, we investigated the effects of *PDE6H* knockout and PDE6 inhibition on tumour growth in vivo.

#### *PDE6H knockout represses HCT116-tumour growth *in vivo

**Fig. 7:**
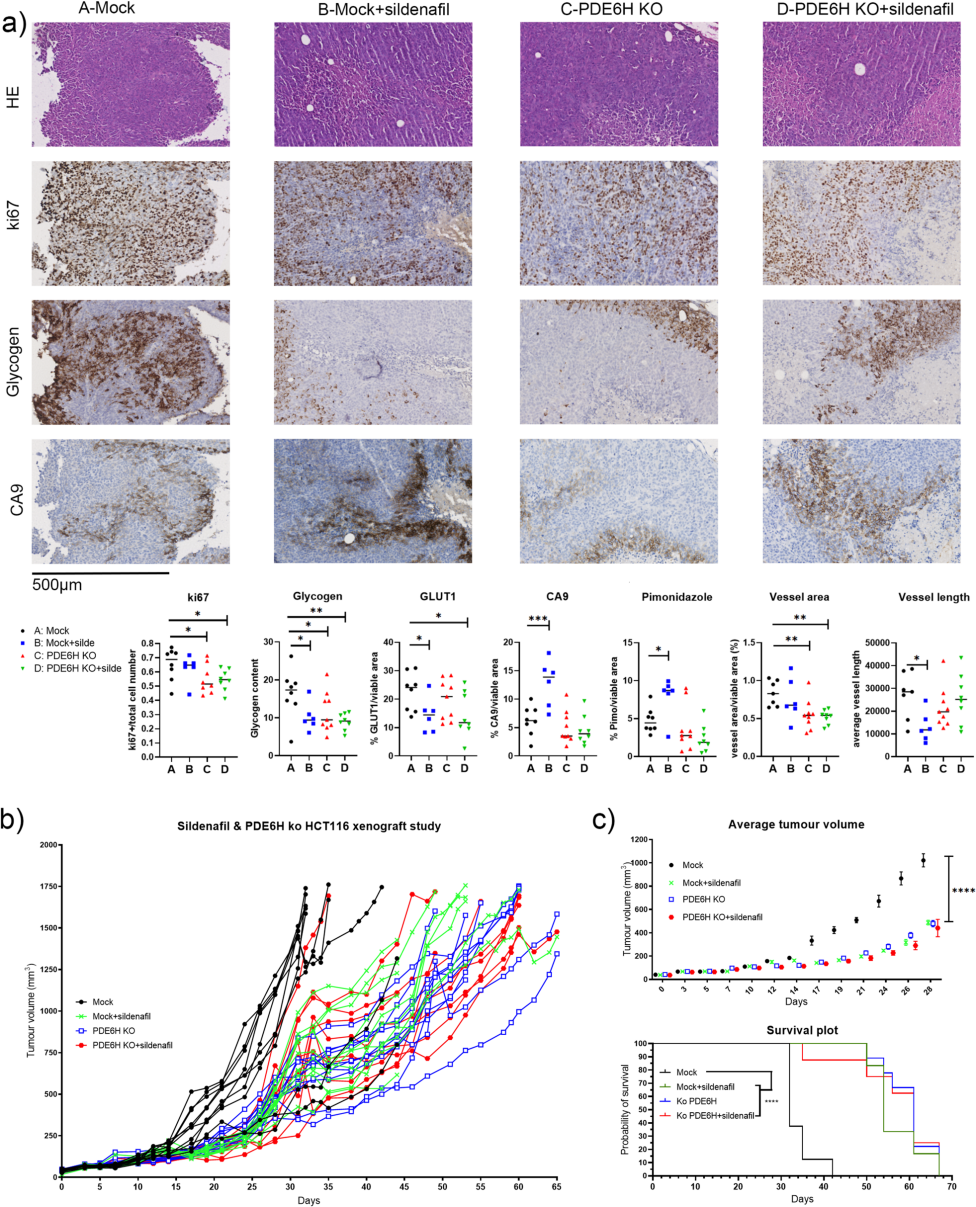
*PDE6H* knockout and sildenafil treatment effect on tumour in HCT116 xenograft model. **a** IHC analysis for markers for proliferation and hypoxia signalling. **b **Tumour growth curves of ctrl- and sildenafil-treated mice of mock or *PDE6H* KO HCT116 xenograft models.* c *Average tumour volumes of treatment groups on day 28 and median survivals of treatment groups. All subjects are shown in* b*, while welfare culls are omitted from **c** in accordance with the criteria described in “Methods”. *p < 0.05, **p < 0.01, ***p < 0.001, ****p < 0.0001

In xenografts, CRISPR-Cas9 mock control and *PDE6H* KO HCT116 were treated with vehicle (corn oil) or 150 mg/kg sildenafil citrate, starting the day the tumour size reached 50 mm^3^. *PDE6H* knockout and sildenafil treatment slowed tumour growth and improved survival (Fig. 7).

Immunohistochemistry (IHC) analysis (Fig. 7a) showed that the glycogen content of *PDE6H* KO HCT116 tumours was lower than those of mock HCT116 tumours. Sildenafil treatment lowered GLUT1 expression in HCT116 mock tumours, supported by a previous finding that sildenafil lowered hypoxia-elevated *Glut1* levels in rat lung [72]. Sildenafil-treated mock HCT116 tumours (B) had higher levels of CA9 protein and pimonidazole stain which are markers for chronic and short-term hypoxia, respectively. Ki-67, glycogen content, and vessel area were lower in *PDE6H* KO HCT116 tumours with (D) and without (C) sildenafil treatment. Taken together with the in vivo and in vitro observations that *PDE6H* KO HCT116 has lower levels of glycogen, while the hypoxic glycogen levels of KO and mock HCT116 are similar, we propose that the KO HCT116 has higher levels of glycogen turnover than mock HCT116. *GLUT1* mRNA levels were lower in *PDE6H* KO HCT116 compared to mock (Fig. 5b). Similarly, sildenafil-treated mock tumours show a decrease in glycogen content and GLUT1.

Sildenafil was expected to have effects on stroma and vessels [29], whereas the effects of *PDE6H* depletion would be confined to tumour cells in KO tumours. Despite this, the effects of sildenafil treatment and *PDE6H* knockout on tumour growth were similar (Fig. 7b). Additionally, the vessel perfusion is higher in the KO and sildenafil-treated groups compared to the control group, at similar levels (Figure S5). When maximal reliable suppression of PDE6γ′ is reached in the KO tumours, further effects of increased perfusion may be difficult to detect. The systemic effects of sildenafil treatment being less pronounced in *PDE6H* KO tumours might help explain why tumour growth is similar in these tumours with or without sildenafil treatment.

## Discussion

In this study, we demonstrate that *PDE6H* plays an essential role in regulating proliferation and metabolism in several cancer cell lines that represent a range of tumour types. We propose that *PDE6H* depletion inhibits PDE6 activity and raises cGMP levels, resulting in disruption to nucleotide biosynthesis and downregulation of mitochondrial activity, G1 arrest, and subsequently cell death.

We identified *PDE6H* as part of a screen to determine metabolic genes which regulate the cell cycle (Fig. 1). Along with other genes that govern both energy production and DNA synthesis, *PDE6H* was one of the G1/S modulators. TCA cycle, fatty acid metabolism, nucleotide metabolism, and DNA repair were shown to be especially important during the transition into S phase, whereas enzymes of electron transport chain, glycolysis, and pentose phosphate pathway were significant for the completion of both G1 and G2 phases. G2/M targets were also populated by genes encoding anabolic enzymes.

DNA synthesis is a high-energy demanding process [73, 74]. Therefore, genes encoding enzymes that facilitate reactions that fuel the cell were expected to regulate G1/S stage, prior to or during DNA synthesis. Processes that increase the nucleotide pools in the cell were also expected to be amongst G1 targets. As its depletion lowers OXPHOS and disrupts nucleotide production, *PDE6H* knockdown and knockout might have a dual effect that prevents DNA synthesis.

PDE6 family genes are highly expressed in vertebrate photoreceptor cells and pineal gland [11], while the invertebrate *Ciona intestinalis* has a single ancestor ortholog to PDE6 catalytic subunits that are necessary for light perception [75, 76]. In mammals, B, C, and D isoforms of PDE6 are used for vision. They are highly expressed in certain breast cancer cell lines and primary breast cancer [77]. *PDE6H* is on the same chromosome band 12p12 as *KRAS*. There is a significant concurrence of copy-number amplifications of *PDE6H* and *KRAS* in cancer cell lines [78] (CCLE, 2019) and in testicular germ cell cancer (TCGA, Firehose Legacy).

While gene expression studies suggest that *PDE6H* is expressed differentialy in disease, protein expression results for PDE6γ′ are absent due to a lack of a commercially available cone gamma-specific antibody. Even the most specific antibody, which shares the least homology with rod gamma by targeting the N-terminal of the protein where PDE6γ′ amino acid sequence diverges from that of PDE6γ, is still likely to detect rod PDE6γ, as half of the epitope consists of an amino acid sequence shared between PDE6γ and PDE6γ′. Additionally, the N-terminal of PDE6γ′ is highly disordered. Developing antibodies against disordered peptides provides a challenge, as such regions have multiple interactions with multiple partners due to their intrinsic flexibility and lack of stable tertiary structures [79].

PDE6γ structure and physiological function have been widely studied. However, our understanding of PDE6γ′ localisation and contribution to PDE6 activation, as well as its impact on cGMP, inferred from studies that focus on PDE6γ activation and conformational changes upon cGMP binding in vivo, combined with observations on PDE6γ′ kinetics. Molecular data on PDE6γ′ are lacking and await the resolution of its atomic-level structure [80].

Through its contribution to the complex stability and the communication between GAF and the catalytic domains, PDE6γ has functions beyond its inhibitory role in the PDE6 complex. The N-terminus contributes to the stability and function of the complex by enhancing cGMP binding and providing a binding site for Gαt-GTP that is necessary for transducin activation [80].

The effects of *PDE6H* depletion could be further supported by overexpression of the gene in knockout cells. Epitope tagging of the inhibitory PDE6 could also help but to ensure specific tagging of PDE6γ′ only, and not PDE6γ; the N-terminal of PDE6γ′ needs to be targeted, which has not previously been done [81, 82]. As the crystal structure of the N-terminal of PDE6γ′, despite its known contribution to PDE6 complex formation and activity [83], has not been fully determined, this would be difficult to interpret. This is beyond the remit of the present work.

Further insight to effects of PDE6γ′ on cell survival comes from retinal diseases. Loss-of-function mutations in PDE6γ′ and PDE6γ contribute to retinal degeneration in achromatopsia and retinitis pigmentosa, respectively [84, 85]. A role for PDE6 in apoptosis regulation has also been suggested [10]. Rd1 and rd10 mice are both retinitis pigmentosa models with PDE6β loss-of-function mutations, displaying rapid and slow rod degeneration, respectively [86]. A gain-of-function mutation of IMDPH, which controls the rate-limiting step for GTP synthesis from R5P, also leads to retinitis pigmentosa [87], emphasising the role of cGMP levels in nucleotide homeostasis. HK2 activity [88] and mTORC1 activation [89] are important for cone survival in rd1 retina.

In vivo inhibition of PDE6γ via AAV-mediated ribozyme knockdown in mice results in reduced photoreceptor function and death, similar to RP with PDE6 loss of function [90]. As retinitis pigmentosa is also a metabolic disorder, the effect in photoreceptors of PDE6 inhibition on nucleotide and energy metabolism has been studied.

The retina’s metabolic demands are governed by light stimuli. Photoreceptors in dark retina consume high levels of ATP to repolarize the plasma membrane [91], while light periods are dominated by biosynthesis to accommodate membrane turnover [92]. Oxygen levels are also lower in dark retina [93] which adapts by switching to aerobic glycolysis [94].

Our LC–MS data showed significant changes to nucleotide levels, indicating a disruption to DNA synthesis upon *PDE6H* deletion and supporting the accumulation of G1 phase in *PDE6H*-depleted cells. *PDE6H* KO HCT116 displayed a similar nucleotide metabolic profile to the dark retina. Similarly, de novo purine synthesis towards GTP and ATP has been shown to be lower in the dark retina with levels of IMP, AMP, and GMP remaining low in dark compared to bright light conditions [95].

We observed an accumulation of DNA precursors, PPP intermediates, glycolysis intermediates, and of purine precursors in *PDE6H* KO HCT116 compared to WT HCT116. There was a depletion of 2-oxoglutarate despite an accumulation of glutamine. LC–MS results suggested that the *PDE6H* KO cells cannot meet the reactant or energy demands of de novo nucleotide synthesis, and that the salvage pathways cannot sustain the nucleotide pools, which helps explain the cell cycle arrest in *PDE6H* depleted cells.

We propose that the decrease in both OXPHOS and glycolysis upon *PDE6H* depletion shown by metabolic rate assays might be due to a preferential utilisation of glucose and glutamine in the PPP, to replenish purine pools (Figure S3a). We hypothesise that in addition to the compensatory entrance of glutamine into TCA cycle, increase in mitochondrial dysfunction upon *PDE6H* knockdown leads to lower OXPHOS (Figure S3b). Although we define consistent metabolic changes and associated protein and mRNA changes, flux analysis would be needed to determine how the metabolic intermediates are processed.

mTORC1 stimulates glutamine-dependent de novo purine production by upregulating the pentose phosphate pathway [96]. Acute purine depletion (e.g. AMP and GMP) [97] and sustained depletion of GTP lead to inhibition of mTORC1 [64]. The lower mTORC1 activity of *PDE6H* depleted cells might account for *PDE6H* KO HCT116 cells being more vulnerable to rapamycin treatment and arginine depletion, for which mTORC1 is a sensor [98]. Sildenafil treatment, through TSC2 phosphorylation by PKG1α, also downregulates mTORC1 activity [99].

Intracellular glycogen levels were also affected by *PDE6H* knockdown, indicated by LC–MS results. While *PDE6H* KO HCT116 cells had lower glycogen in vitro and in vivo, both mock and KO cells rose to similar levels of glycogen in hypoxia. Sildenafil treatment also lowered glycogen levels of mock HCT116 glycogen, while *PDE6H* KO HCT116 cells were not affected. Increased levels of glycogen precursor UDP-gal/glc and gal/glc 1-phosphate, increased mRNA levels of *GYS1*, and decreased levels of glycogen indicate extensive adaptive changes and increased glycogen turnover in *PDE6H* KO HCT116 cells.

The naked mole rat has loss-of-function mutations in cone PDE6 genes, *Pde6c* and *Pde6h*, as revealed by whole genome sequencing compared to other mice [100]. Interestingly, naked mole rat cardiomyocytes share unique features with *PDE6H* depleted cells, such as lower PI3K/AKT pathway activity and lower OXPHOS with higher glycogen turnover [101, 102].

The retina can handle periods of hypoxia and reoxygenation [93]. Adaptation of energy sources alternative to OXPHOS is important to avoiding hypoxic stress and reoxygenation injury. Hypoxia preconditioning via intermittent hypoxia is known to confer cardio protection [103], with glycogen serving as an energy source during ischemia [104].

While our results show a disruption to nucleotide synthesis and energy metabolism, it would be beneficial to identify the key metabolic players in *PDE6H*’s control of the cell cycle. Rescue experiments involving depletion of nutrients, followed by supplementation of WT and *PDE6H* KO cells with glycolysis/TCA cycle/PPP intermediates, could help determine the main effectors of the *PDE6H* depletion phenotype. At the same time, recent transcriptome and metabolite studies of rd1 mice help interpret our results. Gene expression results from the start of rd1 rod degeneration (P10) indicate upregulation of genes related to the catabolic processes of glycolysis, PPP, purine metabolism, OXPHOS, glycogen breakdown, and downregulation of ETC genes in rd1 mice compared to WT [105]. Proteomics results display a downregulation of OXPHOS pathway proteins, supported by morphological abnormalities in mitochondria [105]. Single-cell gene expression of rd1 rod and cones shows that glycolysis and OXPHOS genes are downregulated in cones, while they are upregulated in rods at P17 [106].

cGMP levels govern photoreceptor survival in retinitis pigmentosa models. Inhibition of cGMP-gated channels prolongs rod survival in late-onset disease model rd10 mice and Pde6g-/- mice [107, 108]. Like rd and Pde6g-/- mice, *PDE6H*-deleted HCT116 cells had higher basal levels of cGMP.

While an increase of cGMP is consistently observed upon PDE6 inhibition, specific downstream effectors of the cGMP pathway that regulate the antiproliferative effect are dependent on subcellular cGMP pools. It is a challenge to monitor the spatial control and the systemic effects of a cGMP modulator, as cGMP travels intercellularly. For instance, cGMP produced in cardiac fibroblasts travels to cardiac myocytes via gap junctions [109].

An example of how antiproliferative effect of cGMP pathway effectors are cell-specific come from different mouse models of retinal degeneration. PRKG1 deficiency does not prevent retinal degeneration in Pde6g-/- mice but does so in Cngb1-/- mice, even though cell death in both cases is caused by cGMP elevation [107]. Evidence of cGMP analogues leading to S239 phosphorylation of VASP come from smooth muscle cells and endothelial cells [110]. cGMP elevation acting through PKG/p-VASP might be cell dependant, further signifying the need for cGMP-specific imaging to understand cGMP’s subcellular signalling pools and transport, as the current FRET imaging is not sensitive enough to detect cGMP levels at pM range [111, 112].

A challenge to our study is the multitude of controllers and downstream effectors of cGMP/cAMP signalling [15, 113]. cGMP/PKG pathway has several mechanisms of self-regulation. In cultured smooth muscle cells, treatment with cGMP analogue 8-Br-cGMP reduces PKG1a protein levels by ubiquitinoylation through 26S proteasome activity, as a result of PKG1α autophosphorylation [114]. Additionally, PDE2 and PDE3 are activated and inhibited by cGMP, respectively [115, 116]. This suggests that long-term elevation of cGMP levels might have downstream effects independent of PKG signalling.

We noted the effects of *PDE6H* depletion and elevation of cGMP levels on mitochondrial metabolism likely to be indirect as proof of mitochondrial cGMP signalling is lacking. However, it is known that cGMP signalling affects hypoxia response. Preconditioning rabbit cardiomyocytes by elevated cGMP signalling keeps ischemic necrosis at bay by limiting mitochondrial pore transition through PKG signalling, potentially through its activation of mitochondrial PKC [117, 118]. Mitochondrial cardiomyocyte CMBK channels are the key players of/essential to phosphodiesterase-5 inhibitor/PKG pathway-induced protection from ischemia/reperfusion injury in cardiomyocytes [119]. Their localisation and applicability to other cell lines are still unknown. NO/cGMP/PKG signalling also has cardioprotective effects due to vascular remodelling and neovascularization [120]. Sildenafil treatment is similar to intermittent hypoxic conditioning for increasing blood flow [121].

cGMP signalling has been studied in the context of cancer cell proliferation. Treatment with sildenafil or PKG activation via cGMP analogues affects survival, leading to apoptosis in breast and colorectal cancer cell lines as well as colorectal xenografts [63, 122]. On the other hand, sildenafil potentially promotes melanoma growth by activating MAPK signalling [123]. Increased NOS activity and cGMP levels correlate with higher angiogenesis and lymph node metastasis in head and neck cancer patients [124]. Another cGMP-producing enzyme is GUCY2C; its inactivation is linked to diet-induced obesity and has been suggested to contribute to the onset of colorectal cancer by upregulation of proliferation AKT/mTOR signalling while suppressing mitochondrial function in mouse intestinal epithelial cells and human colorectal cancer cells [125–127].

PDE5/6 inhibitors also affect cell metabolism. Zaprinast lowers pyruvate driven OXPHOS in isolated mice brain mitochondria [67] and GLS activity in pancreatic cancer cells [128]. A 2-h tadalafil treatment of C2C12 skeletal muscle cells decreases activity of TCA enzyme citrate synthase and β-oxidation enzyme 3-hydroxy acyl-CoA dehydrogenase [129].

Sildenafil treatment alleviates insulin resistance [130]. Treatment with ANP-induced cGMP production increases insulin secretion from rat islets, while NO donors and cGMP analogues direct insulin-stimulated GLUT4 translocation and glucose uptake in human vascular smooth muscle cells [131, 132]. In mouse cardiomyocytes exposed to pressure overload, sildenafil treatment downregulates genes of Pi3k-Akt pathway, decreases Akt phosphorylation, improves functional mitochondria levels, and lowers ROS while alleviating myopathy [133]. PDE inhibition might have greater therapeutic potential for diabetes and cancer than cGMP production inducers [134].

## Conclusion

We propose that the growth inhibitory effects of PDE6γ′ inhibition are due to the following:

1. Increase in cGMP levels that induce cell death and mitochondrial dysfunction. We have not observed an increase in PKG activity in *PDE6H* knockdown and HCT116 when we checked the whole cell lysates upon the cGMP increase which suggests either localised increase in PKG activity or an effect of cGMP independent of PKG.
2. Disruption of nucleotide metabolism and cell cycle progression due to accumulation of cGMP, a purine synthesis intermediate, with both these effects potentially lowering mTORC1 activity [64, 135, 136].

Here, we define a role for photoreceptor cone gene *PDE6H* in regulating cancer cell proliferation and metabolism. The results suggest that *PDE6H* would be a new target worth assessing.

## Methods

### Cell lines

HCT116 (colorectal carcinoma) and MDA-MB-436 (breast adenocarcinoma) which were obtained from Muscle Lab stocks and NCI-H23 (non-small cell lung cancer adenocarcinoma) which was a gift of Prof. Andy Ryan were characterised by Eurofins and grown in 1 g/L glucose DMEM (Dulbecco Modified Eagle Medium 11,885, Gibco) which was supplemented with 10% FBS (Fetal Bovine Serum, Gibco), 100 U/mL of penicillin, and 50 g/mL of streptomycin. HCT116 was grown and transfected in 4.5 g/L glucose (11,965) and supplemented with 10% FBS as part of the siRNA screen. Cells were passaged every 4 days using 0.05% trypsin–EDTA.

### siRNA reverse transfection

Lyophilised siRNAs (Dharmacon) were resuspended in 1 × siRNA buffer (Dharmacon) to prepare a stock of 2 μM for siRNA screen and 10 μM for all other transfections. Reverse transfection for the siRNA screen was carried out in flat-bottom 96-well plates using a final concentration of 20-nM siRNA diluted in 20-μL DharmaFECT2 Cell Culture Reagent (DCCR) (Dharmacon), complexed with 0.2-μL DharmaFECT2 transfection reagent (Dharmacon), and mixed with 80 μL of antibiotics-free cell suspension of 10,000 HCT116 cells. Cells were prepared for propidium iodide (PI) staining on the 3rd day of transfection.

Reverse transfection of the cell lines was otherwise carried out with a final concentration of 20-nM siRNA in OptiMEM (Gibco) and 0.2% by volume of DharmaFECT2 for HCT116 and DharmaFECT1 for NCI-H23 and MDA-MB-436. A total of 1:4 ratio of transfection complex to antibiotics-free cell suspension was used. RNA extraction was carried out after 48 h of reverse transfection. All other siRNA transfected sample collections, including those for protein extraction, flow cytometry analysis, or collection of cells for replating, were carried out on the third day of transfection after replacing transfection media with full cell-culture media containing antibiotics after 48 h of transfection.

### RNA isolation and reverse transcription polymerase chain reaction (RT-qPCR)

Cells grown to 80% confluency were lysed directly with TRI Reagent (Thermo Fisher). Chloroform was then added to the cell lysate and mixture shaken for 30 s at room temperature (RT). The aqueous upper phase was mixed with glycogen (Thermo Fisher) in a clean tube, and RNA was precipitated by adding isopropanol through centrifugation. The RNA pellet was centrifuged and washed twice with cold 75% ethanol. Ethanol was then removed and RNA left to dry for an hour in the tissue culture hood, on ice. RNA was resuspended in DEPC-treated water and was dissolved by heating 60 °C for 10 min with intermittent vortexing, before getting quantitated using NanoDrop (Thermo Fisher).

CDNA was synthesised from 1 μg of total RNA treated with DNase I, Amplification Grade (Invitrogen). Annealing step was performed using 5 μM of oligo(dT)_20_ (Thermo Fisher) and 1-mM dNTP (Thermo Fisher) through a 5-min incubation at 65 °C, followed by a 1-min incubation at 4 °C. The reverse transcriptase master mix was prepared with 4 μL 5 × SSIV buffer, 1-μL 100-mM DTT, 1-μL RNAout ribonuclease inhibitor, and 1-μL SSIV reverse transcriptase (Thermo Fisher) as per manufacturer’s instructions. Reverse transcriptase reaction was carried out by incubating the mixture at 55 °C for 10 min and then at 80 °C for 10 min. RT-qPCR reaction was carried out at a volume of 20 μL, using 400 nM of primer mix and with a final 1 × concentration of SYBR No-ROX mastermix (SensiMix Bioline). PCR procedure was carried out using a QuantStudio 5 Real-Time PCR instrument, with 1 cycle of 55 °C for 2 min and 95 °C for 10 min, 40 cycles of 95 °C for 15 s, 58 °C for 30 s, and a final melting curve stage of 95 °C for 15 s, 58 °C for 1 min, and 95 °C for 15 s.

### CRISPR/Cas9 nickase stable knockout cell line generation

Plasmids were prepared as per Zhang Lab protocol [137] based on PX461: SpCas9n-2A-EGFP (D10A nickase) and PX462: SpCas9n-2A-Puro (D10 nickase). HCT116 cells were transfected with Lipofectamine 3000 reagent (Thermo Fisher) complexed with P3000 reagent and 2 μg of each plasmid dissolved in OptiMEM, a day after seeding. Transfection efficiency was determined using ZOE Fluorescent Cell Imager (Bio-Rad) to be 70% 24 h after transfection. Puromycin selection (1.5 μg/mL) was carried out for 2 days after which the remaining cells were incubated in complete culture media for a day. Cells were then suspended and replated in a 0.5 cells/well concentration to obtain single-cell plating. After 10 days, cells that grew into single colonies were replated and expanded in 6-well plates up to 80% confluency. The resulting clones were tested via genomic DNA sequencing and PCR product visualisation to confirm target deletion. Pools of mock control cells were obtained by transfection with plasmids without the sgRNAs and were confirmed for WT *PDE6H* status.

### Genomic DNA purification

Cells were lysed with DNAzol (ThermoFisher) and DNA precipitated by inversion after adding 100% ethanol and a 2-min incubation at RT. DNA precipitate was obtained in a clean tube by spooling with a pipette tip and washed twice with 75% ethanol. Ethanol was removed, and DNA was dissolved in 8-mM NaOH. A total of 500 ng DNA was amplified in a 50-μL reaction volume including 0.2-mM dNTP mix, additional 3-mM MgCl_2_ GoTaq-DNA polymerase (Promega), colourless GoTaq® reaction buffer, and 1-μM “PDE6H gen” forward (fwr) and reverse (rev) primers. PCR thermal cycling protocol was carried out as 2 min at 95 °C; followed by 40 cycles of 30 s each at 95 °C, 58 °C, and 72 °C; and a final cycle of 5 min at 72 °C.

### Protein immunoblotting

Adherent cells were washed with PBS and lysed with 1 × RIPA buffer (Sigma) containing protease inhibitor cocktail (Roche) and phosphatase inhibitor mini tablets (Pierce, Thermo Fisher) and obtained in Eppendorf tubes. Lysates were shaken on an orbital shaker for 30 min at 4 °C and then centrifuged 30 min at 4 °C and 13,000 rpm to remove the cell debris. Protein concentration was determined via DC protein assay (Bio-Rad). Samples were prepared by heating 20 μg of total protein with 1 × NuPAGE LDS Sample Buffer (Invitrogen) and 1 × NuPAGE Sample Reducing Agent at 90 °C for 5 min. Gel electrophoresis was carried out using NuPAGE 4–12% Bis–Tris, 1.5-mm (Invitrogen) protein gels for 1 h at RT under constant voltage of 100 V in 1 × NuPAGE MES SDS Running Buffer (Invitrogen). Gels were transferred to 0.45-μM pore size Immobilon-P PVDF Membrane (Merck) using TE62 transfer cooled unit (GE Healthcare) under constant voltage of 55 V at 4 °C in 1 × NuPAGE Transfer Buffer solution containing 20% methanol. Membranes were allowed to dry for an hour at RT following transfer. They were then rewetted, washed with 1 × TBS, and blocked for an hour using 5% BSA (bovine serum albumin) (A9647, Sigma) in TBS. Primary antibody solutions contained 0.1% Tween 20 (Sigma) in 1 × TBS (TBST), with 5% BSA in which the blots were incubated overnight at 4 °C. Secondary incubation of membranes was conducted with HRP-linked antibodies (7074 and 7076) in a 5% BSA TBST solution for an hour at RT. Membranes were washed with TBST four times for 5 min each. Reblotting of membranes was performed by incubating membranes in Restore PLUS Western Blot Stripping Buffer at RT for 25 min, followed by washes and an hour of blocking, before reprobing with another primary antibody. Reblotted images of the same membrane were presented together in a single frame. Each membrane was blotted for β-actin separately.

### Flow cytometry

#### High-throughput propidium iodide (PI) staining for cell cycle siRNA screen

Transfected cells in 96-well plates were trypsinised and obtained in wells of a V-bottom plates containing old growth media. Next, cell media were discarded following centrifugation, and cells were resuspended and incubated in 50 μL of 1:1 PBS:Accumax (Sigma-Aldrich) solution for 15 min at RT. Cells were then fixed by adding the suspension dropwise to a fresh v-bottom 96-well plate containing 130 μL/well of ice-cold 100% ethanol and incubated at 4 °C for 90 min. Fixed cells were washed with cold PBS and resuspended in 100 μL of PI solution in PBS containing 200 μg/mL RNAse A (Sigma-Aldrich) and 50 μg/mL PI solution (Sigma-Aldrich). Following an incubation at 4 °C for 30 min, DNA content analysis via flow cytometry was carried out based on FL2 laser readings using FACSCalibur (BD Biosciences). Cell cycle distribution histograms were drawn using FlowJo software. FSC-H vs SSC-H plots were used for gating cells. A second gate was applied on FL2-W vs FL2-A graph to omit doublets. The propidium iodide (PI) staining was utilised for determining if the cell population of interest has a DNA content of “n” or “2n”. The percentage of cells with “n” amount of DNA corresponds to population of cells in G1/S phase, and the percentage of cells with “2n” amount of DNA indicated the cells in G2/M phase. The percentage increases in percentage of cells in G1/S or G2/M stages from siNT values were then used to calculate a Z-score for each siRNA. Z-scores were based on the standard deviation of G1/S or G2/M percentages of siNT transfected cells across different plates. A high Z-score indicated a significant arrest at the stage of interest.

#### Detailed analysis of cell cycle distribution

Adherent cells were incubated with 10-μM EdU solution of Alexa Fluor 647 Click-iT EdU Flow Cytometry Assay Kit (Thermo Fisher) for 2 h. Cells were washed, lifted via trypsinisation, resuspended in fresh media, and obtained in round-bottom 96-well plates. Media were discarded after centrifugation, and cells were then incubated in 50 μL 1:1 PBS:AccuMax mix for 15 min at RT to obtain a single-cell suspension. Cells were fixed overnight by adding 100-μL fixation/permeabilisation solution (BD Biosciences) to the cell suspension and incubating overnight at 4 °C, protected from light. Fixed cells were washed with 1% BSA PBS and incubated in Click-iT reaction cocktail as per manufacturer’s protocol, for 30 min at RT. After a washing step in 1% BSA in 1 × Perm/Wash Buffer, cells were incubated for 1 h in p-Histone3 primary antibody and then for 30 min in secondary antibody (Alexa Fluor 555 anti-mouse secondary, 1:2000). After a washing step in 1% BSA in PBS, cells were suspended in 1 × FxCycle Violet stain solution and 5 μL/mL anti-ki67 antibody solution (BioLegend, FITC) in PBS and were ready for analysis after a 30-min incubation. Cells were analysed via FACS Attune (Thermo Fisher) VL1 (FxCycle), BL1 (Ki-67), YL1 (p-Histone3), RL1 (BrDU).

As part of to flow cytometry analysis, apoptotic/necrotic cell populations were indicated by cells with subG1 (< n) DNA content determined by FxCycle. Amongst cells with ≥ n DNA content, Ki67 − cells made up the quiescent, and Ki67 + cells made up the proliferating cell population. Ki67 + cells were grouped according to their DNA content determined by FxCycle, BrDU, and p-Histone3 signals. BrDU cells with “n” DNA made up the G1 population, the BrDU + “n-2n” cells made up S. BrDU − cells with “2n”, p-Histone3 − cells made up G2 population, while BrDU − , “2n”, and p-Histone3 − cells made up M population. The data were expressed as percentage of cells in each cell cycle stage relative to the total number of total or viable cells.

#### Cell death

Adherent cells were trypsinised and prepared as single cell suspension in U-bottom 96-well plates as described in “Detailed analysis of cell cycle distribution”. Annexin V + PI solution was added to the cell suspension to make up final concentrations of 5 μL/mL of Annexin V (BioLegend, FITC), 200 μg/mL RNAse A (Sigma-Aldrich), and 50 μg/mL PI solution (Sigma-Aldrich) in 1 × PBS. PI + cells denoted late apoptotic/necrotic cells, PI − Annexin V + cells early apoptotic, and PI − Annexin V-cells live cell populations. Cells were analysed via FACS Attune (Thermo Fisher) BL1 (Annexin V), YL2 (PI).

#### Cleaved caspase-3 and LipidTOX

Adherent cells were trypsinised, prepared as single-cell suspension, and fixed in U-bottom 96-well plates as described in “Detailed analysis of cell cycle distribution”. Fixed cells were washed with 1% BSA (bovine serum albumin) PBS and incubated for 1 h in cleaved caspase-3 primary antibody and then for 30 min in secondary antibody (Alexa Fluor 488 goat Anti-Rabbit Secondary, 1:2000). Cells were analysed via FACS Attune (Thermo Fisher) BL1 (Annexin V), YL2 (LipidTOX).

#### Mitochondria dyes

As part of mitochondrial ROS measurement, adherent cells were incubated at 37 °C for 30 min with DMSO-dissolved stock MitoSOX (Invitrogen) added to culture media at a final concentration of 5 μM. Cells were then trypsinised and prepared as single-cell suspension in U-bottom 96-well plates as described. A total of 1:1 PBS:AccuMax solution was removed following centrifugation and resuspended in PBS. Cell suspension was analysed via FACS Attune (Thermo Fisher) BL2 laser.

MitoTracker Deep Red vs MitoTracker Green plots were used to determine populations of cells with dysfunctional mitochondria (deep red − , green +) that have lost their membrane potential [138]. Adherent cells were incubated at 37 °C for 45 min with DMSO dissolved MitoTracker Deep Red (Invitrogen) and MitoTracker Green (Invitrogen) added to culture media at final concentrations of 30 nM and 20 nM, respectively. The resulting cell pellet was resuspended in 1-mL PBS and analysed via FACS Attune (Thermo Fisher) BL1 (Green) and YL2 (Deep Red) laser after cells were gated using FSC-A vs SSC-A plots.

#### Seahorse metabolic analysis

Cells were grown for overnight in 96-well Seahorse XF plates (Agilent) in the following densities: HCT116 22,500, NCI-H23 35,000, and MDA-MB-436 35,000 cells/well. On the day of the assay, cells were washed twice and supplied with pH = 7.4 serum-free assay media before being placed in the non-CO_2_ incubator for 1 h. Mitochondrial stress test assay media consisted of 5-mM glucose, 1-mM pyruvate, 4-mM glutamine, and XF base medium; drugs used were oligomycin (1 μM), FCCP (0.5 μM), and antimycin (0.5 μM). Glycolysis stress test assay media consisted of 4 mM glutamine and XF base medium; supplement/drugs used were glucose (100 mM), oligomycin (1 μM), and 2-DG (500 mM).

Following Seahorse assay, cell media was then removed, and Seahorse plates were placed in − 80 °C. CyQUANT viability test was conducted to normalise assay results to DNA content. Cells were thawed and were lysed through pipetting after being suspended in a solution of cell-lysis buffer and CyQUANT GR dye which was prepared in Milli-Q water. Cells were incubated for 10 min at RT and protected from light, and their fluorescence signal readings were then taken using a fluorescence microplate reader at excitation/emission wavelengths 485 nm/520 nm.

#### Sample preparation for GC–MS analysis

Cells were washed with ice-cold PBS and scraped after being incubated for 1 min in 80% ice-cold methanol. Cells were lysed by pipetting on ice. Cell debris was removed by centrifuging at 13,000 rpm for 30 min at 4 °C. Supernatant was then filtered using a 10-kD molecular weight cut-off filter (Amicon Ultra) to remove soluble protein from the metabolite solution. DNA concentration of the samples was measured using NanoDrop (Thermo Fisher). Filtrates were adjusted in volume using 80% methanol to obtain the same concentration of total DNA across samples. Samples were analysed using LC–MS to determine mass to charge ratios (m/z) and their retention times to identify metabolite composition and abundances through “IC-MS” (ion-exchange chromatography mass spectrometry), “RPLC-MS” (reversed-phase chromatography), and “RPLC-MS” with derivatisation’ methods [65, 139].

#### Spheroid generation

Cells were prepared into single-cell suspensions and seeded at volume of 100 μL in a 96-well spheroid microplate with a round-bottom low-attachment surface (4515, Corning) before being centrifuged for 10 min at 2000 rpm at RT. Seeding density was 4000 cells/well for HCT116 and 5000 cells/well for NCI-H23 and MDA-MB-436. Following an overnight incubation, wells were topped up with fresh media or media containing sildenafil. Four days after seeding, spheroids started to form. Spheroids were imaged with EVOS FL cell imaging system (ThermoFisher). Total and necrotic spheroid areas were calculated using Adobe Photoshop.

#### Xenograft tumour growth

A total of 2.5 × 10^6^ HCT116 cells in 200 μL of Matrigel (BD Bioscience) were implanted subcutaneously into female CD1 nude mice. Tumour growth was monitored and measured using calipers and tumour volume calculated using the formula V = L × W × H × π/6. Sildenafil citrate was administered at a volume of 250 μL at 150 mg/kg (Merck, PHR1807) dissolved in corn oil (Sigma Aldrich, C8267) via oral gavage, 5 days a week (Mon-Fri) until the tumour volume reached 1500 mm^3^ or at the endpoint of the experiment. Pimonidazole (2 mg/kg) and 647-tomato lectin (1 mg/kg)/Hoescht (2 mg/kg) mix was given intravenously 5 min before the mice were culled after perfused tumours were fluorescence imaged using the IVIS system.

#### Immunohistochemistry

Paraffin-embedded 10-μm tissue sections were dewaxed by incubating at 60 °C for 10 min, followed by two RT incubations in Histo-Clear solution (National Diagnostics), for 5 min each. Sections were rehydrated through consecutive immersions twice in 100% IMS, followed by 70% IMS, 50% IMS, and Milli-Q water, for 5 min each. Antigen retrieval was performed by boiling the slides for 2 min in a pressure cooker, immersed in 1 × target retrieval solution, pH = 6 (Dako, Agilent). Tumour regions were sealed using hydrophobic Dako Pen (Agilent). Sections were incubated in peroxidase-blocking solution (Dako, Agilent) for 15 min and washed with wash buffer. Sections were blocked by applying 10% horse serum in PBS containing 1 drop/5 mL of mouse-on-mouse blocking reagent (Vector Labs) for 40 min. Sections were incubated overnight at 4 °C in primary antibody diluted in antibody diluent (Flex, Agilent). They were then washed in wash buffer, three times for 5 min, on the roller. The secondary antibody conjugate Envision Flex/HRP (Dako, Agilent) was applied for 20 min, followed by another set of washes. DAB solution in Flex substrate buffer was applied for 5 min to visualise the antibody.

Nuclei were stained by applying FLEX Hematoxylin (Dako, Agilent) for 10 min. Haematoxylin and eosin staining was carried out on dewaxed and rehydrated sections by incubating the samples in haematoxylin solution for 1 min, followed by Milli-Q wash, 30 s in Scott’s tap water substitute, a second Milli-Q wash, 10 s in acetyl acid supplemented eosin solution (Sigma-Aldrich, 109,844), and a final Milli-Q wash. After staining, slides were dried via a 1-h incubation step at 40 °C. Sections were mounted with cover slips using Entellan Mounting Medium (Merck). Slides were imaged using Akoya Biosciences Vectra Polaris Automated Quantitative Pathology Imaging System. Sections were scored using machine-learning trained custom Visiopharm applications (Visiopharm).

#### Immunofluorescence

Cells grown in μ chamber slides (Ibidi) were PBS washed and fixed with 4% PFA for an hour at RT. After PBS wash, cells were permeabilised and blocked in 10% horse serum and 0.1% triton in PBS. They were then incubated overnight at 4 °C in primary antibody solution (1:200 glycogen, gift of Otto Baba) in 10% horse serum in PBS. After three washes with PBS, cells were incubated in the secondary solution in PBS (1:1000, Alexa Fluor 555 goat anti-mouse). Slides were mounted with VECTASHIELD Antifade Mounting Medium with DAPI (Vector Labs) and imaged using Zeiss 880 LSM confocal microscope.

#### Glycogen content assay

After the media of adherent cells were removed, cells were obtained in ice-cold PBS via scraping and mixed by gentle pipetting. The cell suspension was centrifuged at 5000 rpm for 10 min and placed in − 80 °C overnight. Thawed samples were boiled at 100 °C for 10 min and spun at 15,000 rpm for 15 min to remove insoluble material. The glycogen content of the supernatant was measured as per the fluorometric assay protocol of BioVision Glycogen Assay Kit.

#### cGMP ELISA

Media was removed from adherent cells which were incubated with 0.1-M HCl for 30 min at RT. Cells were then scraped and homogenised by pipetting and then centrifuged at 2000 rpm at 4 °C for 10 min. Samples and HCl-dissolved cGMP standards were acetylated as per manufacturer’s instructions (Cayman). Samples were incubated in the ELISA plate for 18 h at 4 °C and protected from light. Development was carried out at room temperature, protected from light, for 90 min, using an orbital shaker. Absorbance reading of the plate was taken at 405 nm, and concentrations were calculated using Cayman’s online ELISA analysis tool: (https://www.caymanchem.com/analysisTools/elisa).

#### Clonogenic survival assays

Cells were seeded at the density of 400 cells/well. A day after seeding, cells were treated with rapamycin or sildenafil. Cells were refed with arginine depleted or arginine containing media 2 days after seeding. Clonogenic survival assays of arginine-depleted cells were conducted in SILAC media (Gibco, A2493901) supplemented with 5-mM glucose, 1-mM sodium pyruvate (Sigma Aldrich, S8636), 4-mM glutamine (Agilent, 103,579–100), phenol red (Sigma-Aldrich, P0290), and lysine-HCl (Sigma-Aldrich, 444,208) to emulate arginine-free DMEM (11,885). Medium for control groups of arginine depletion clonogenic assay was prepared by adding arginine-HCl (Sigma-Aldrich 08163). After about 10 days when visible colonies defined as cluster have formed, plates were scanned and colonies counted using ImageJ.

#### Quantification and statistical analysis

Multiple *t*-test analysis was conducted when comparing the means between two groups based on one independent variable, such as in RT-qPCR, mass spectroscopy analyses (*FDR* = 1%), and flow cytometry analyses between siNT and siPDE6H, except for cell cycle data. One-way ANOVA was used to compare more than two groups based on one variable, such as IHC analyses of tumour sections. Two-way ANOVA analyses were conducted to compare groups based on multiple independent variables, such as all other flow cytometry analyses, as well as those of clonogenic survival assays, Seahorse metabolic assay components, glycogen enzyme assay, and spheroid size calculations.

## Supplementary Information

**Additional file 1: Supplementary figures S1-S4**.

**Additional file 2: Table S1.** Custom siRNA library.

**Additional file 3: Table S2.** Normalised percentage differences in G1, S and G2 populations of G1/S and G2/M hits from Run 3 of the screen, as well as hits that have caused an unnormalized increase of >5% in G1 or G2 populations.

**Additional file 4: Table S3.** Mass spectroscopy results showing metabolite abundances normalised to the mean of all samples.

**Additional file 5: Table S4:** List of abbreviations.

## Data Availability

Data generated or analysed during this study are included in this published article and its supplementary information files. Further details are available from the corresponding author on reasonable request.
