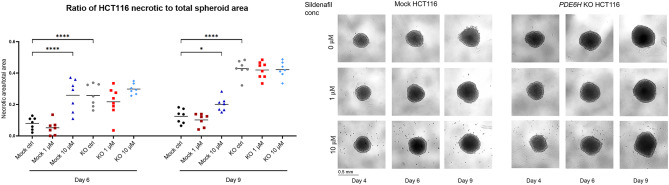# Correction to: Cone photoreceptor phosphodiesterase *PDE6H* inhibition regulates cancer cell growth and metabolism, replicating the dark retina response

**DOI:** 10.1186/s40170-024-00371-1

**Published:** 2025-01-28

**Authors:** Ceren Yalaz, Esther Bridges, Nasullah K. Alham, Christos E. Zois, Jianzhou Chen, Karim Bensaad, Ana Miar, Elisabete Pires, Ruth J. Muschel, James S. O. McCullagh, Adrian L. Harris

**Affiliations:** 1 Molecular Oncology Laboratories, Department of Medical Oncology, Weatherall Institute of Molecular Medicine, John Radcliffe Hospital, University of Oxford, Oxford, OX3 9DS UK; 2 Department of Engineering Science, Institute of Biomedical Engineering (IBME), University of Oxford, Old Road Campus Research Building, Oxford, OX3 7DQ UK; 3 Department of Oncology, University of Oxford, Old Road Campus Research Building, Oxford, OX3 7DQ UK; 4 Department of Chemistry, University of Oxford, Mansfield Road, Oxford, OX1 3TA UK

**Correction to: Cancer Metab 12, 5 (2024).**

10.1186/s40170-023-00326-y

In the original publication of this article the middle row of Fig. 6e was inadvertently duplicated during figure assembly. The calculations were performed using batch analysis, so graph remains accurate and the conclusions are unaffected. The incorrect and correct figure are shown in this correction article, the original article has been updated.

**Incorrect figure**.


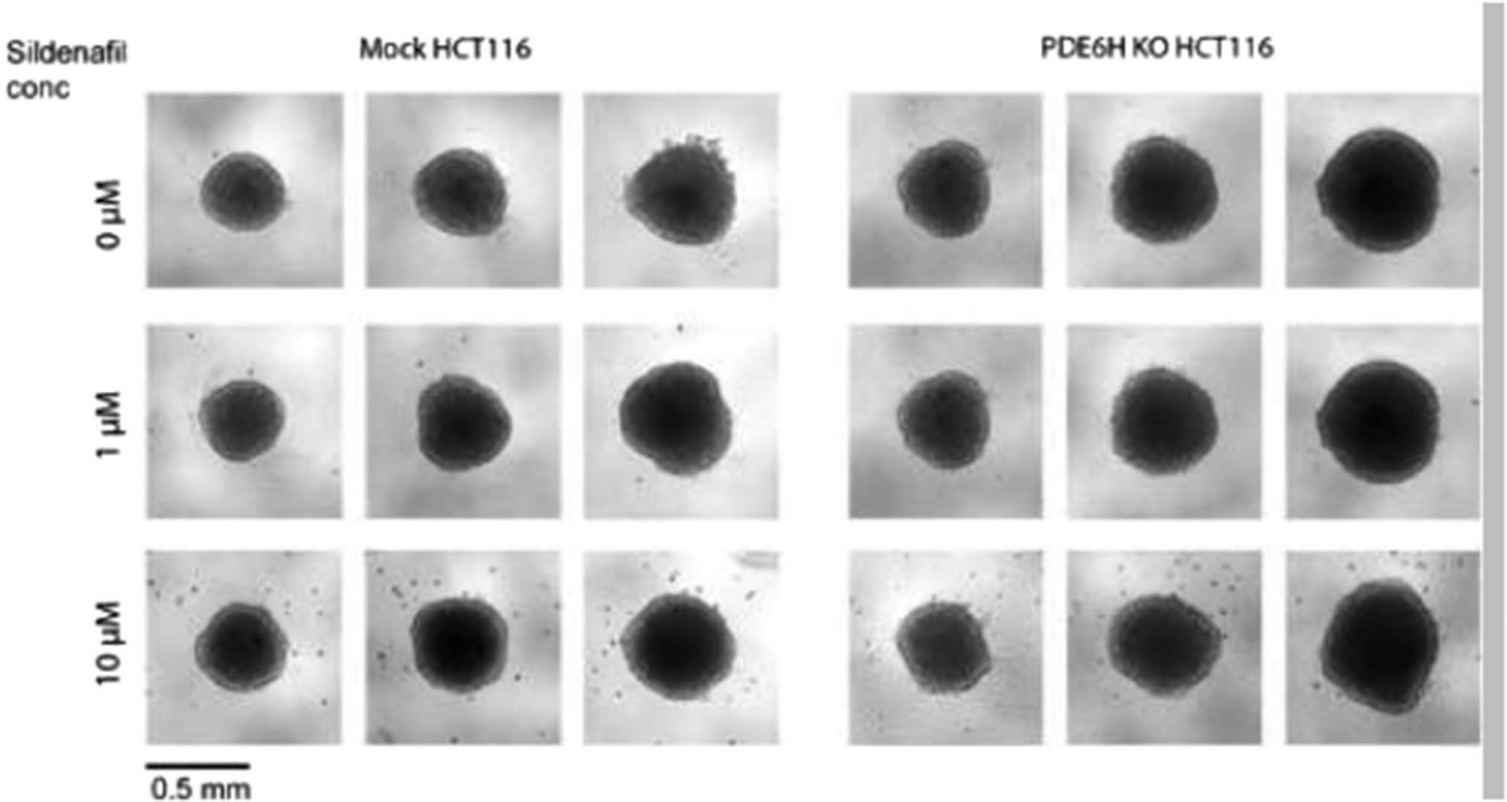


**Correct figure**.