# Supplementary material for: Cone photoreceptor phosphodiesterase PDE6H inhibition regulates cancer cell growth and metabolism, replicating the dark retina response
Source: Cancer Metab. 2024 Feb 13;12:5. doi: 10.1186/s40170-023-00326-y (PMC10863171; doi:10.1186/s40170-023-00326-y)
Supplement: Supplementary file 1 — Additional file 1: Supplementary figures S1-S4. [file 40170_2023_326_MOESM1_ESM.zip › Supplementary clonogenic survival.pptx]

## Slide 1
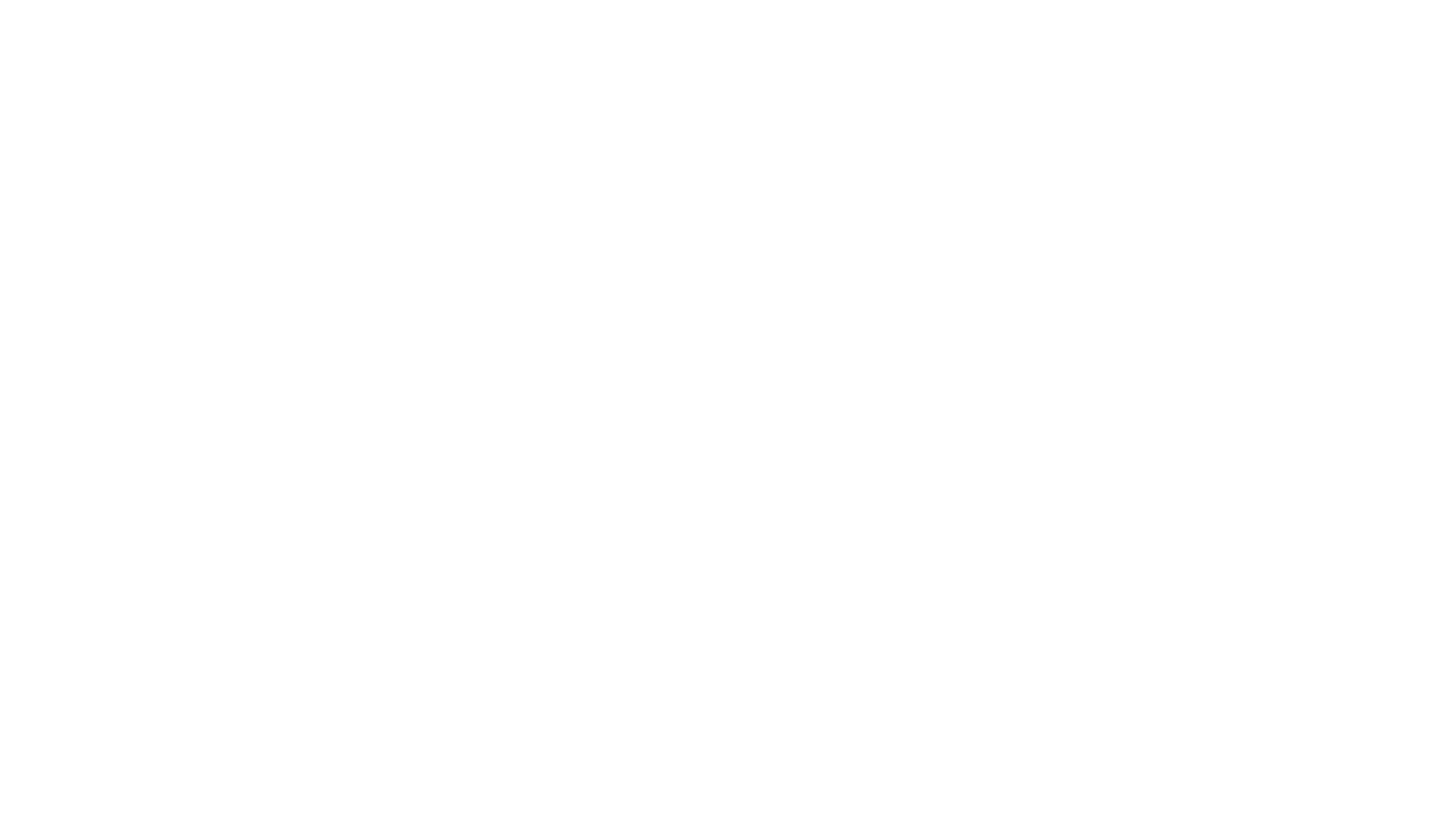

#

## Slide 2
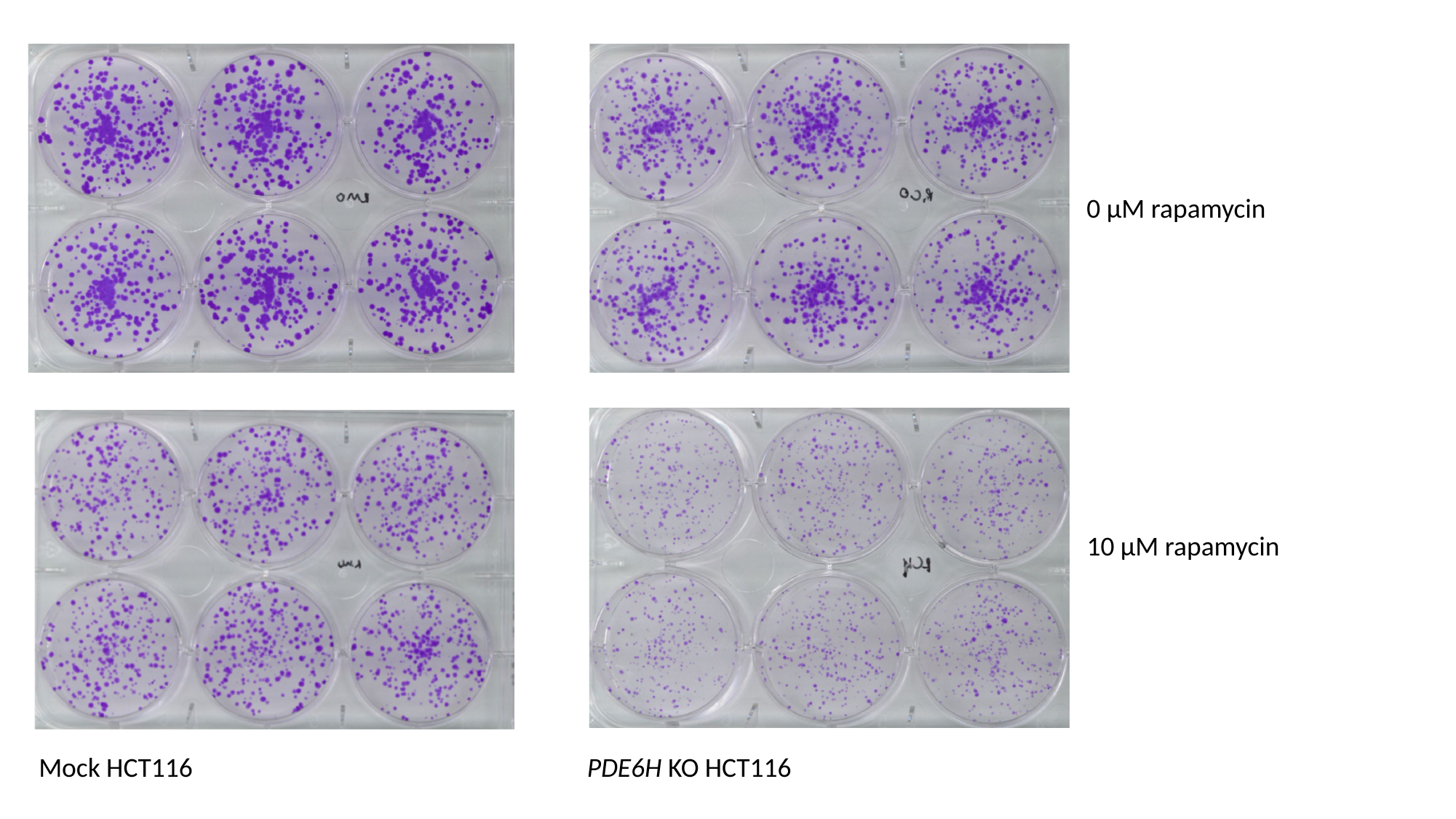

#
0 μM rapamycin
10 μM rapamycin
Mock HCT116
PDE6H KO HCT116

## Slide 3
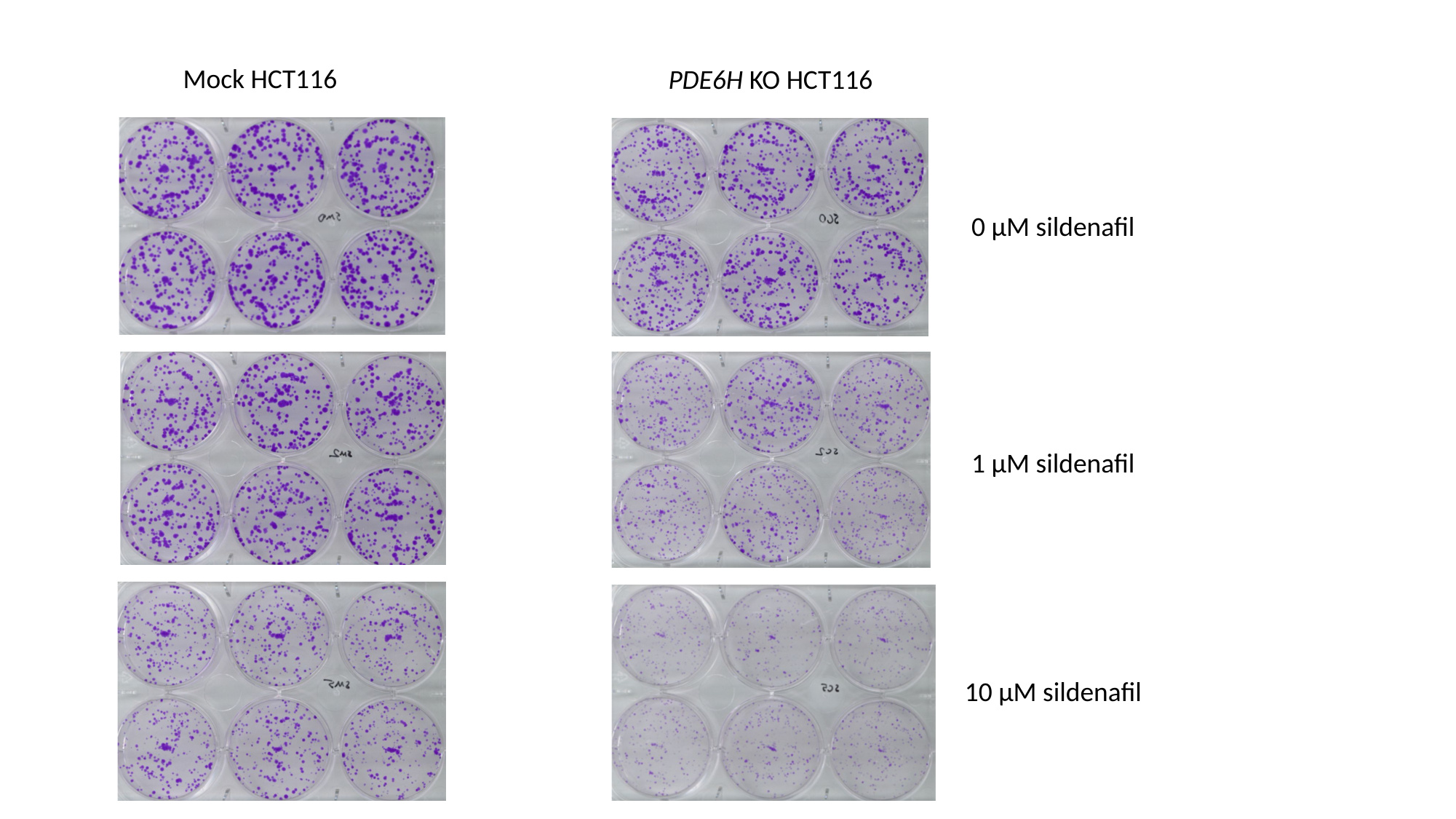

Mock HCT116
PDE6H KO HCT116
0 μM sildenafil
1 μM sildenafil
10 μM sildenafil

## Slide 4
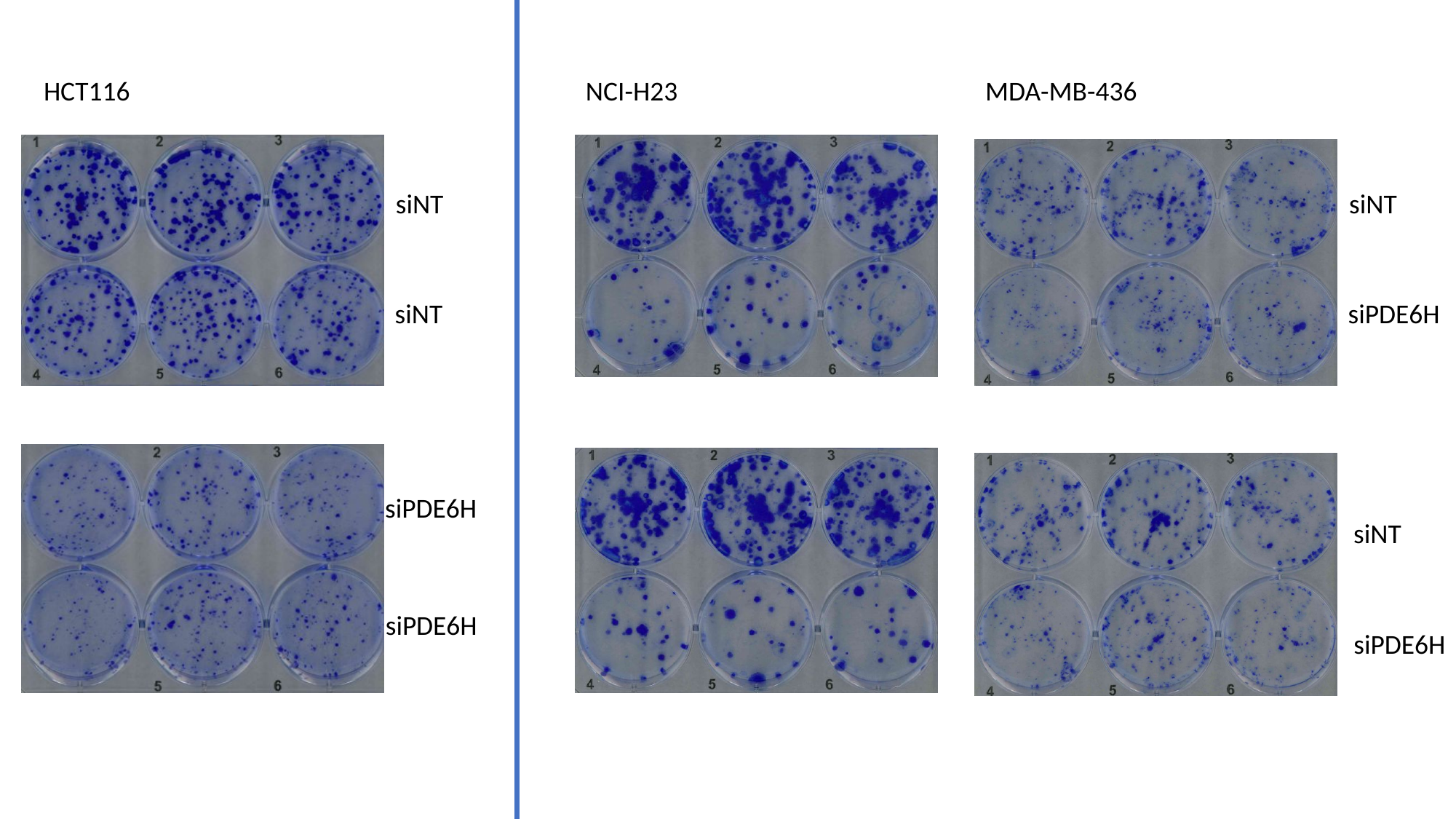

HCT116
NCI-H23
MDA-MB-436
siNT
siNT
siNT
siPDE6H
siPDE6H
siNT
siPDE6H
siPDE6H

## Slide 5
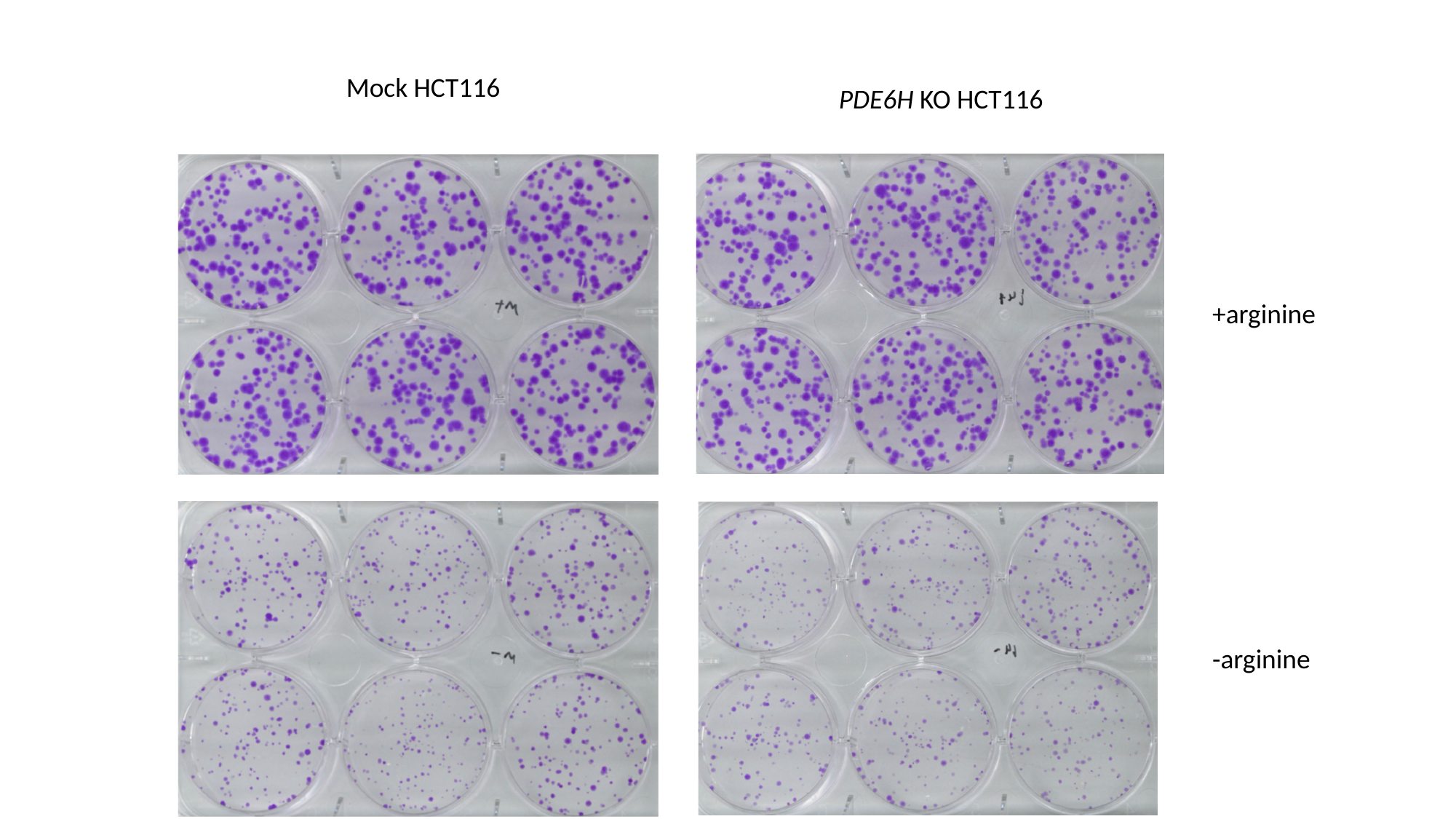

Mock HCT116
PDE6H KO HCT116
+arginine
-arginine
