# Supplementary material for: Cone photoreceptor phosphodiesterase PDE6H inhibition regulates cancer cell growth and metabolism, replicating the dark retina response
Source: Cancer Metab. 2024 Feb 13;12:5. doi: 10.1186/s40170-023-00326-y (PMC10863171; doi:10.1186/s40170-023-00326-y)
Supplement: Supplementary file 1 — Additional file 1: Supplementary figures S1-S4. [file 40170_2023_326_MOESM1_ESM.zip › Supplementary information_vs4.docx]

**Supplementary tables**

Table S1: Custom siRNA library

Table S2: Normalised percentage differences in G1, S and G2 populations of G1/S and G2/M hits from Run 3 of the screen, as well as hits that have caused an unnormalized increase of >5% in G1 or G2 populations.

Table S3: Mass spectroscopy results showing metabolite abundances normalised to the mean of all samples, obtained via ‘IC-MS’ (ion-exchange chromatography-mass spectrometry), ‘RPLC-MS’ (Reversed-phase chromatography), and ‘RPLC-MSd’ (reversed-phase chromatography with derivatisation) (Walsby-Tickle et al., 2020).

Table S4: List of abbreviations


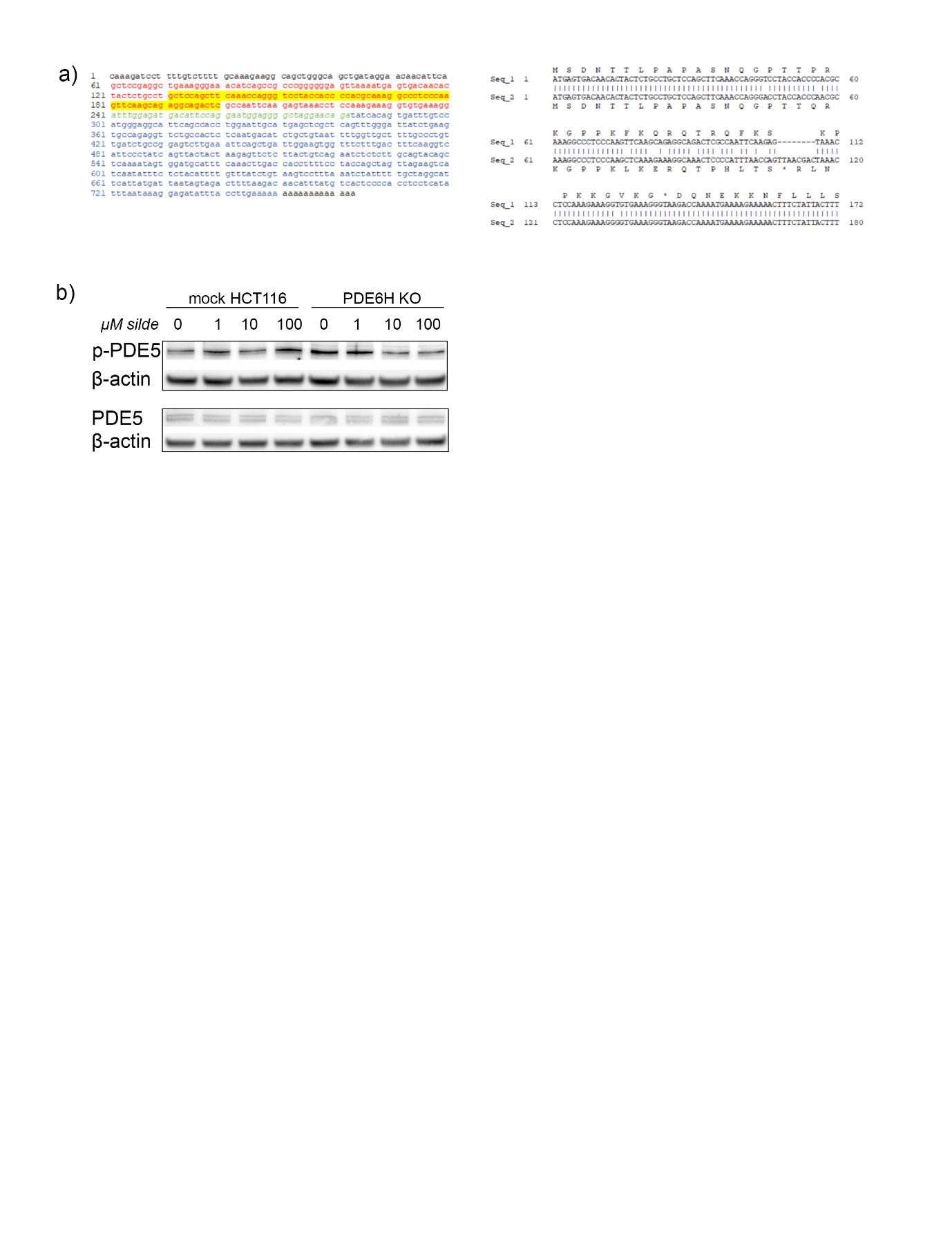
**Supplementary figures**


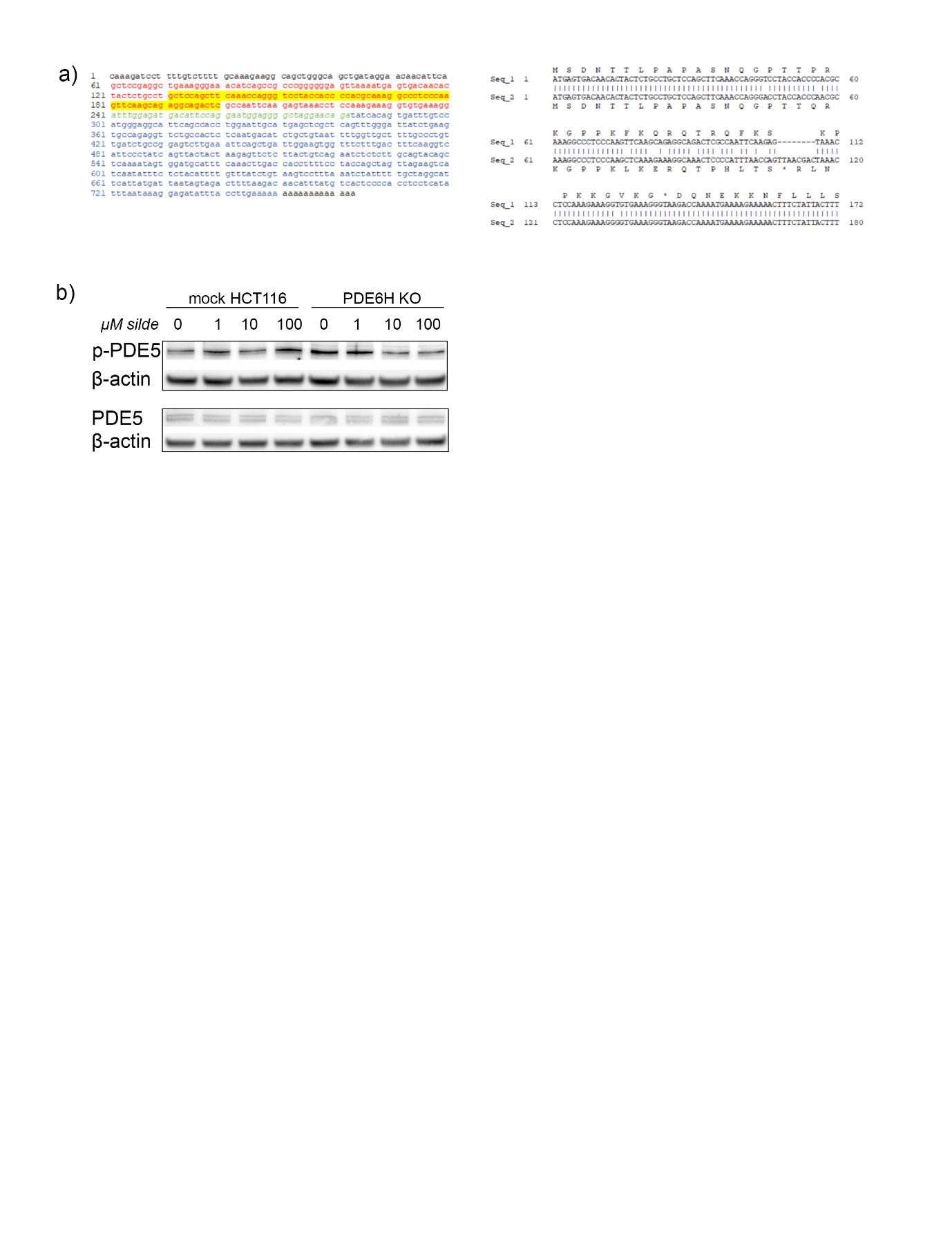

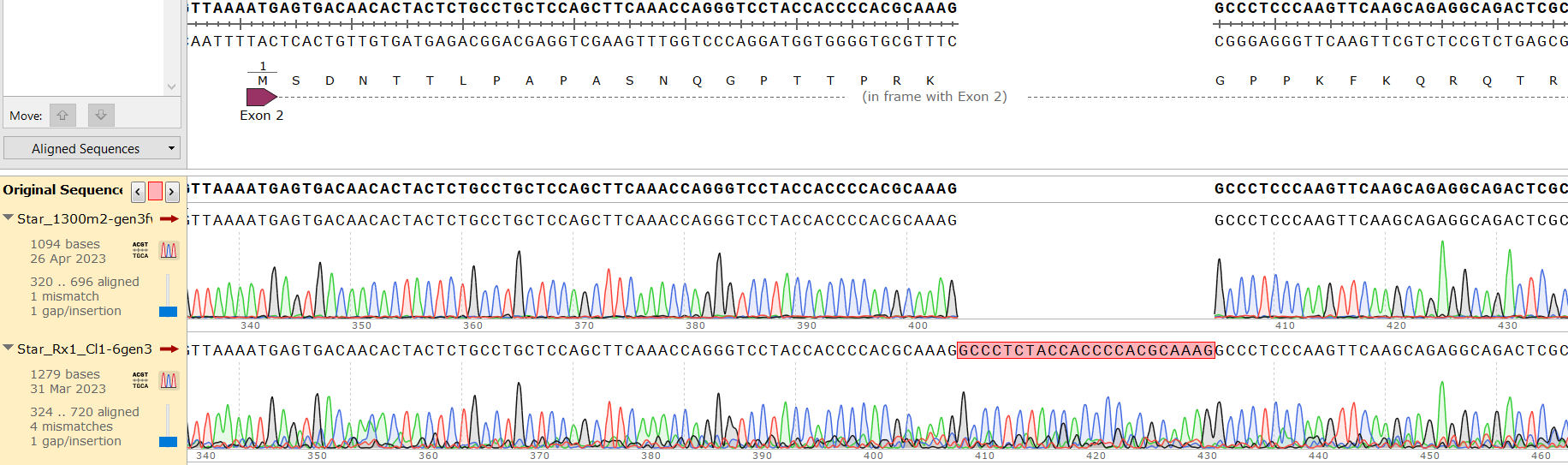


Figure S1 (Related to Figure 3): a) Sequences of *PDE6H* mRNA (Section of exon 2 targeted by CRISPR-Cas9 is highlighted, corresponding to aa 9-30 of *PDE6H*) and sequence chromatograms for the mock control HCT116 and *PDE6H* KO HCT116 genomic DNA, in alignment with the *PDE6H* sequence, are presented. *PDE6H* KO included of 23 base pairs that resulted in a frameshift mutation from aa 26 to the rest of exon 2 (aa 45). Aa 4 and 13-14 of *PDE6H* enhance activation by transducin (Wang et al., 2019). Aa 10-30 enhances allosteric cGMP binding to GAF domain (Mou and Cote, 2001) (Zhang et al., 2012). cGMP binding to PDE6 GAF domain enhances PDE6γ binding to the complex (Pugh and Lamb, 1993) (Mou and Cote, 2001). Aa 17-31 of *PDE6H* include three threonine residues; the two Thr residues in rod *PDE6G* in the same region have been shown to be phosphorylated upon light activation (Janisch et al., 2009). Lysine rich region aa 21-41 promotes PDE6H binding to the catalytic site; aa 73-83 interacts with the PDE6 catalytic site (Mou and Cote, 2001). N-terminus of *PDE6H* also sequesters cGMP bound to the GAF domain until a cGMP threshold is reached (Gulati et al., 2019).

b) P-PDE5 levels of mock HCT116 increased with sildenafil treatment and were basally lower than those of *PDE6H* KO HCT116.

Figure S2 (Related to Figure 3): cGMP levels of HCT116 KO compared to mock control following treatment with 10 μM sildenafil citrate for 10 days (n=4) were measured. Error bars represent standard deviation (* p<0.05, ** p<0.01, **** p<0.0001).

Figure S3 (Related to Figure 4): Levels of key metabolites for which the WT and *PDE6H* KO trends mimic those in light and dark retina, respectively. Relative changes between *PDE6H* WT and KO HCT116 metabolites were presented as log_2_ values of the metabolite abundances normalised to average values of all samples. Error bars represent standard deviation, p<0.05.


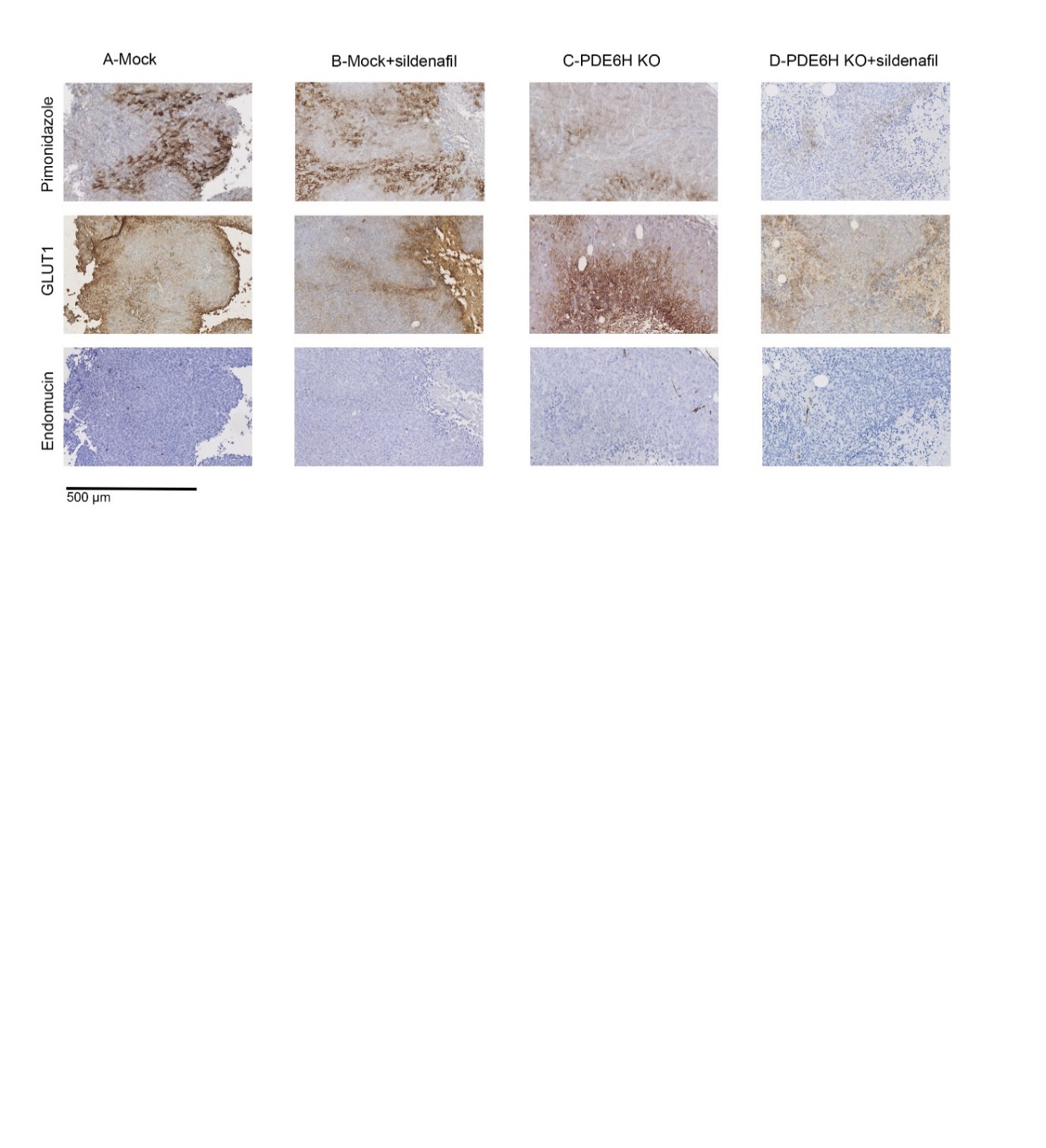


Figure S4 (Related to Figure 7): Representative images of IHC staining for GLUT1, pimonidazole and endomucin.

Figure S5 (Related to Figure 7): Perfusion levels of PDE6H deleted tumours and tumours of mice treated with sildenafil were higher than those of the ctrl group. * p<0.05, ** p<0.01.


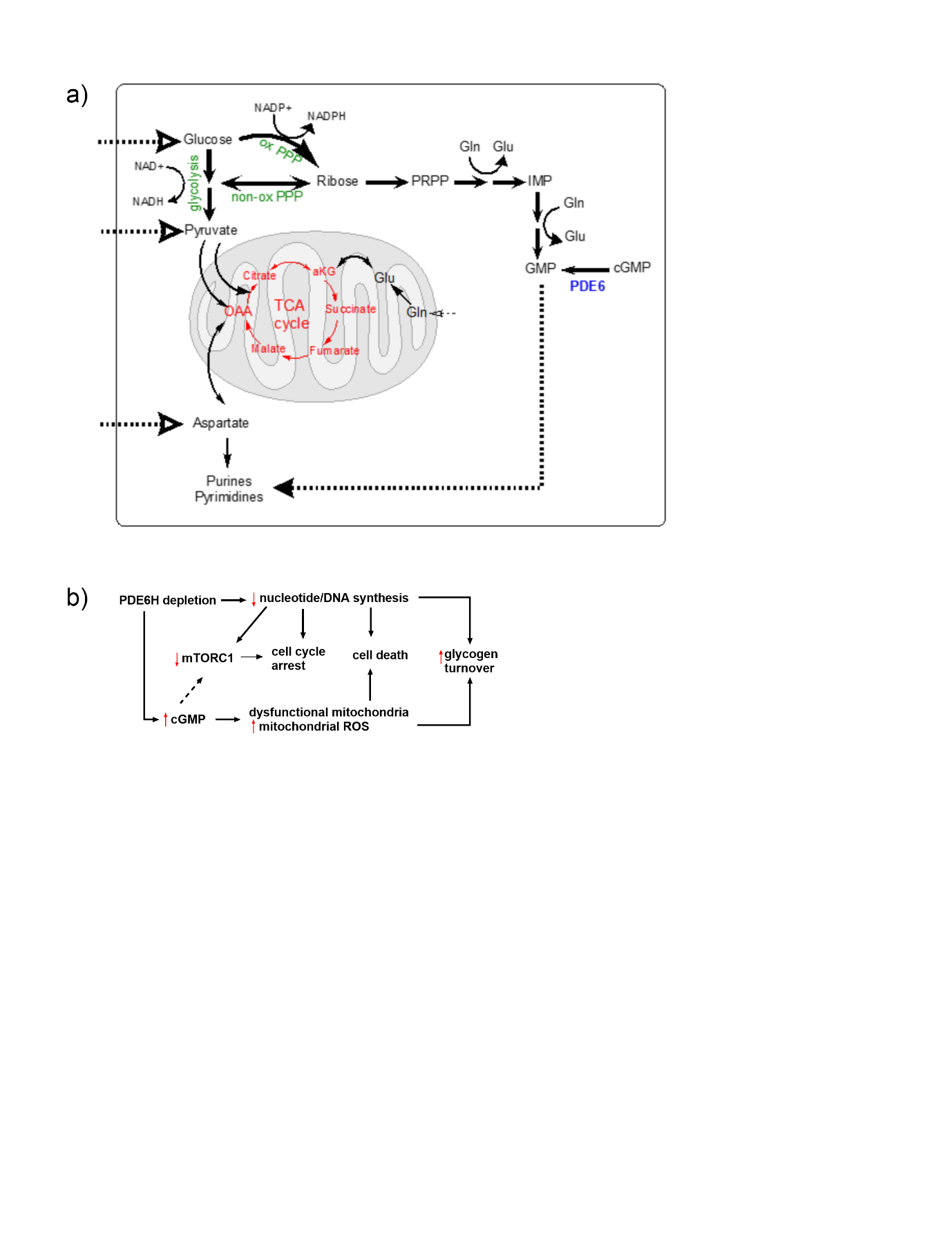


Figure S6: a) Hypothetical rerouting of glutamine in *PDE6H* depleted cells. b) Summary of effect of *PDE6H* depletion on cell proliferation and metabolism.

**SI references**

Gulati, S., Palczewski, K., Engel, A., Stahlberg, H., and Kovacik, L. (2019). Cryo-EM structure of phosphodiesterase 6 reveals insights into the allosteric regulation of type I phosphodiesterases. Sci Adv *5*, eaav4322. 10.1126/sciadv.aav4322.

Janisch, K.M., Kasanuki, J.M., Naumann, M.C., Davis, R.J., Lin, C.S., Semple-Rowland, S., and Tsang, S.H. (2009). Light-dependent phosphorylation of the gamma subunit of cGMP-phophodiesterase (PDE6gamma) at residue threonine 22 in intact photoreceptor neurons. Biochem Biophys Res Commun *390*, 1149-1153. 10.1016/j.bbrc.2009.10.106.

Mou, H., and Cote, R.H. (2001). The catalytic and GAF domains of the rod cGMP phosphodiesterase (PDE6) heterodimer are regulated by distinct regions of its inhibitory gamma subunit. J Biol Chem *276*, 27527-27534. 10.1074/jbc.M103316200.

Pugh, E.N., and Lamb, T.D. (1993). Amplification and kinetics of the activation steps in phototransduction. Biochim Biophys Acta *1141*, 111-149. 10.1016/0005-2728(93)90038-h.

Walsby-Tickle, J., Gannon, J., Hvinden, I., Bardella, C., Abboud, M.I., Nazeer, A., Hauton, D., Pires, E., Cadoux-Hudson, T., Schofield, C.J., and McCullagh, J.S.O. (2020). Anion-exchange chromatography mass spectrometry provides extensive coverage of primary metabolic pathways revealing altered metabolism in IDH1 mutant cells. Commun Biol *3*, 247. 10.1038/s42003-020-0957-6.

Wang, X., Plachetzki, D.C., and Cote, R.H. (2019). The N termini of the inhibitory γ-subunits of phosphodiesterase-6 (PDE6) from rod and cone photoreceptors differentially regulate transducin-mediated PDE6 activation. J Biol Chem *294*, 8351-8360. 10.1074/jbc.RA119.007520.

Zhang, X.J., Gao, X.Z., Yao, W., and Cote, R.H. (2012). Functional mapping of interacting regions of the photoreceptor phosphodiesterase (PDE6) γ-subunit with PDE6 catalytic dimer, transducin, and regulator of G-protein signaling9-1 (RGS9-1). J Biol Chem *287*, 26312-26320. 10.1074/jbc.M112.377333.
