# Supplementary material for: Cone photoreceptor phosphodiesterase PDE6H inhibition regulates cancer cell growth and metabolism, replicating the dark retina response
Source: Cancer Metab. 2024 Feb 13;12:5. doi: 10.1186/s40170-023-00326-y (PMC10863171; doi:10.1186/s40170-023-00326-y)
Supplement: Supplementary file 1 — Additional file 1: Supplementary figures S1-S4. [file 40170_2023_326_MOESM1_ESM.zip › Supplementary WB.pptx]

## Slide 1
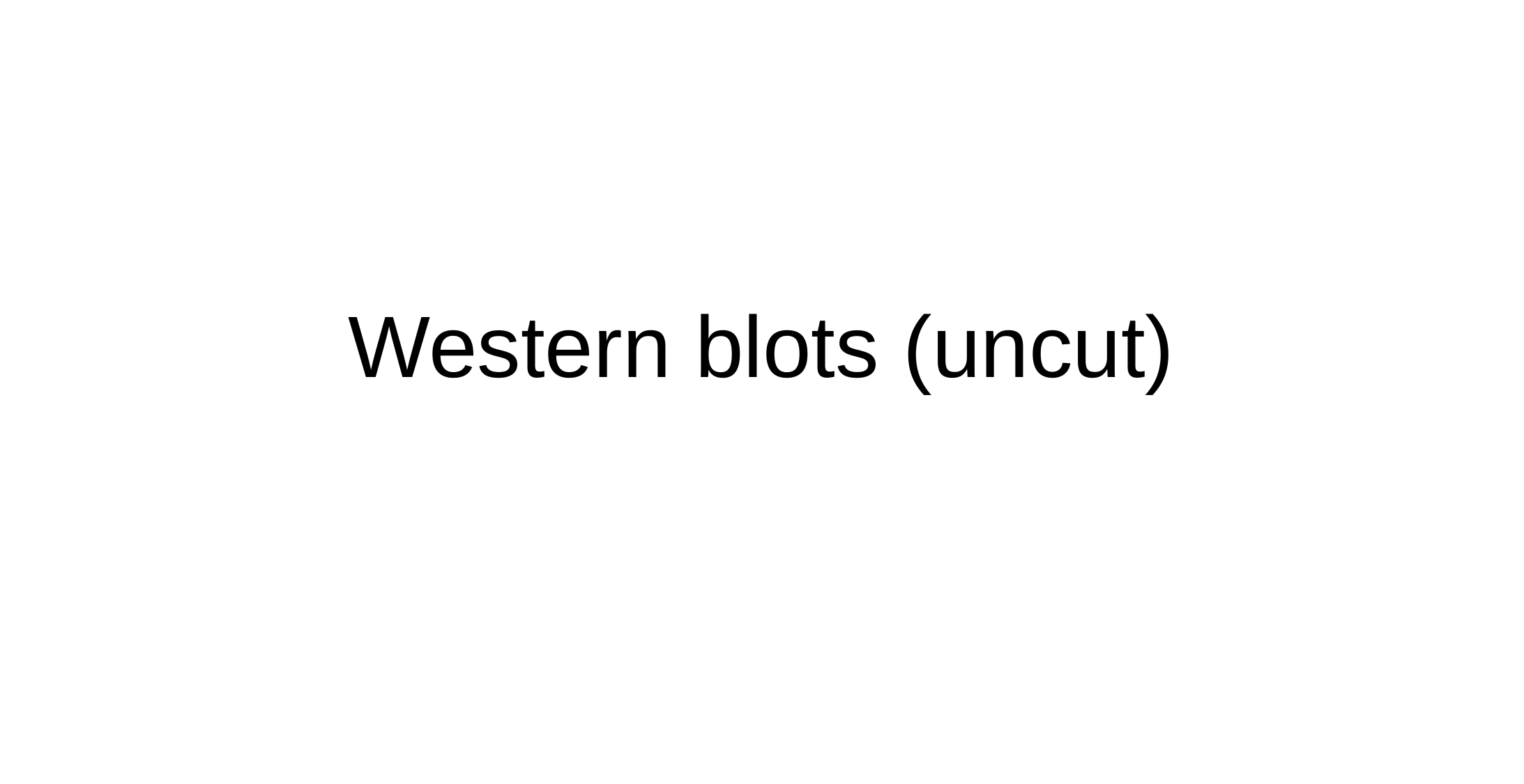

# Western blots (uncut)

## Slide 2
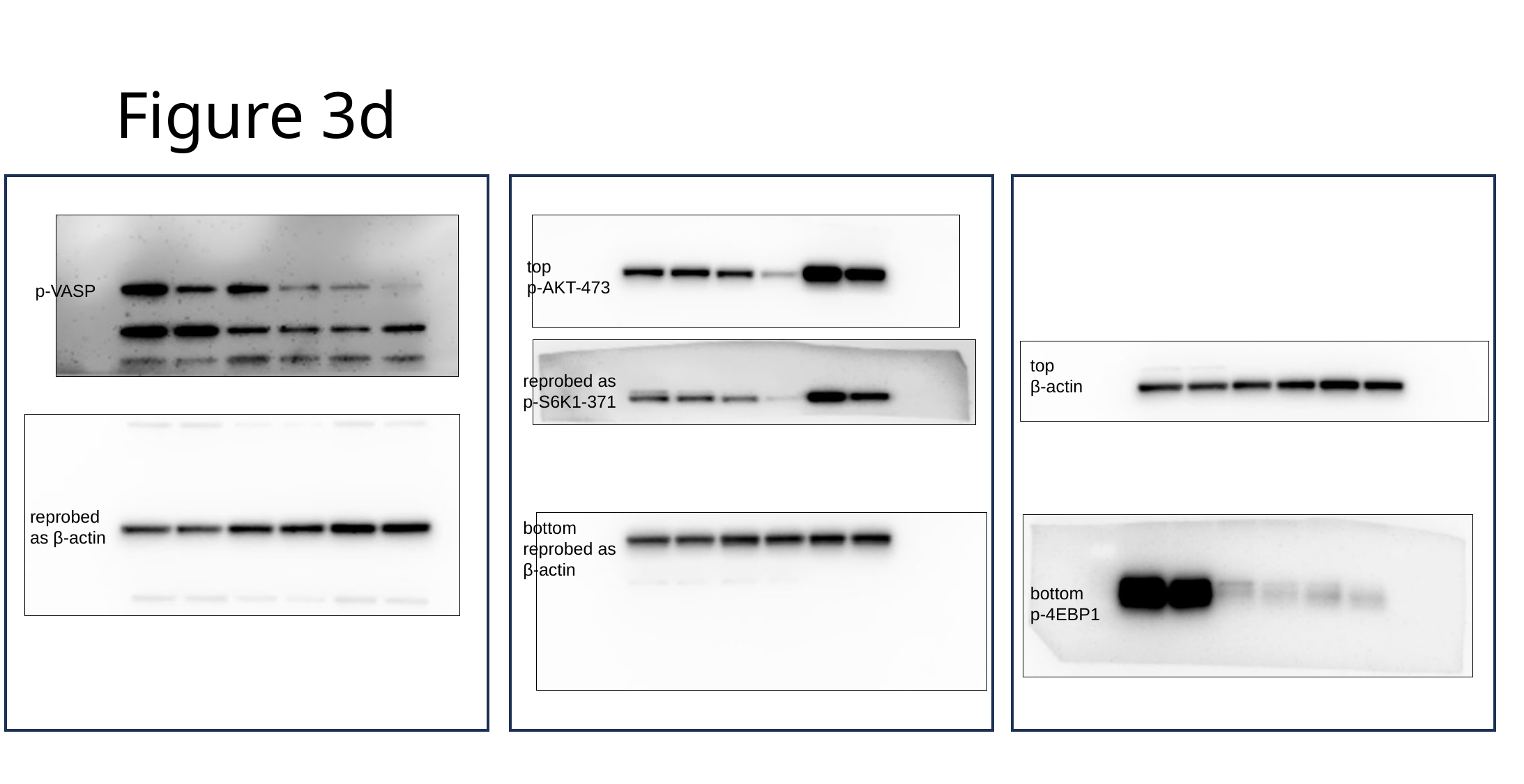

# Figure 3d
top
p-AKT-473
p-VASP
top
β-actin
reprobed as
p-S6K1-371
reprobed as β-actin
bottom reprobed as
β-actin
bottom
p-4EBP1

## Slide 3
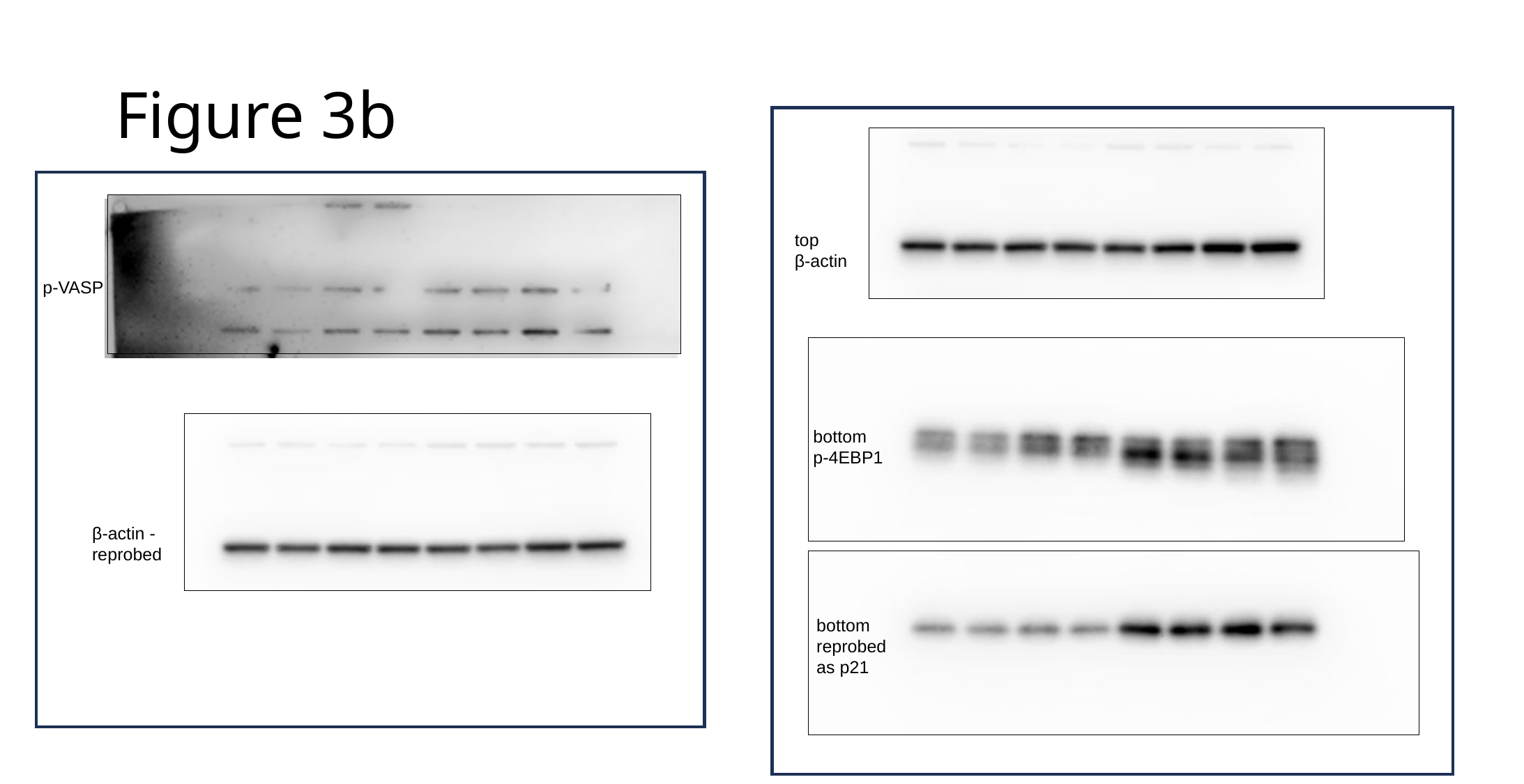

# Figure 3b
top
β-actin
p-VASP
bottom
p-4EBP1
β-actin -reprobed
bottom reprobed as p21

## Slide 4
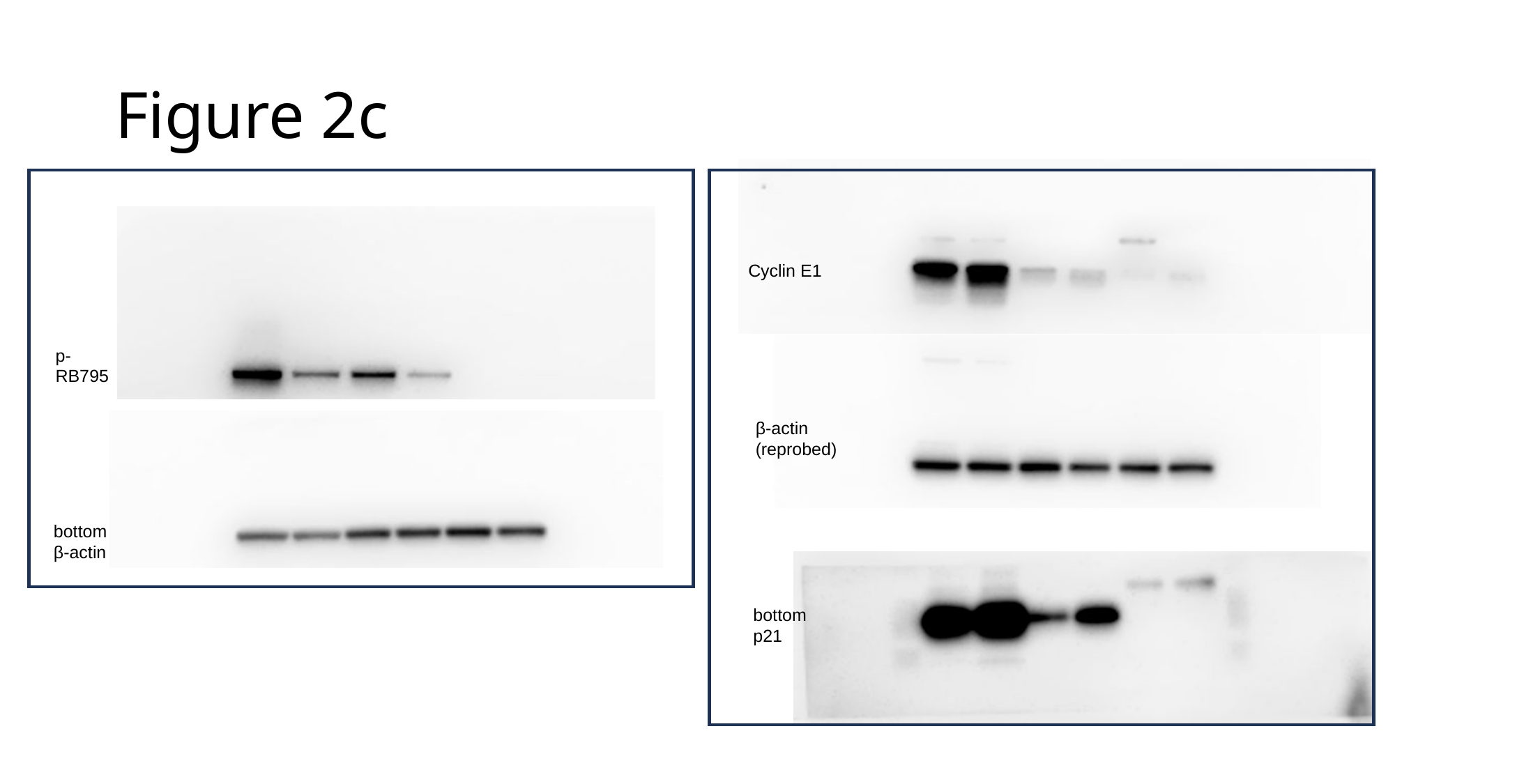

# Figure 2c
Cyclin E1
p-RB795
β-actin (reprobed)
bottom
β-actin
bottom
p21

## Slide 5
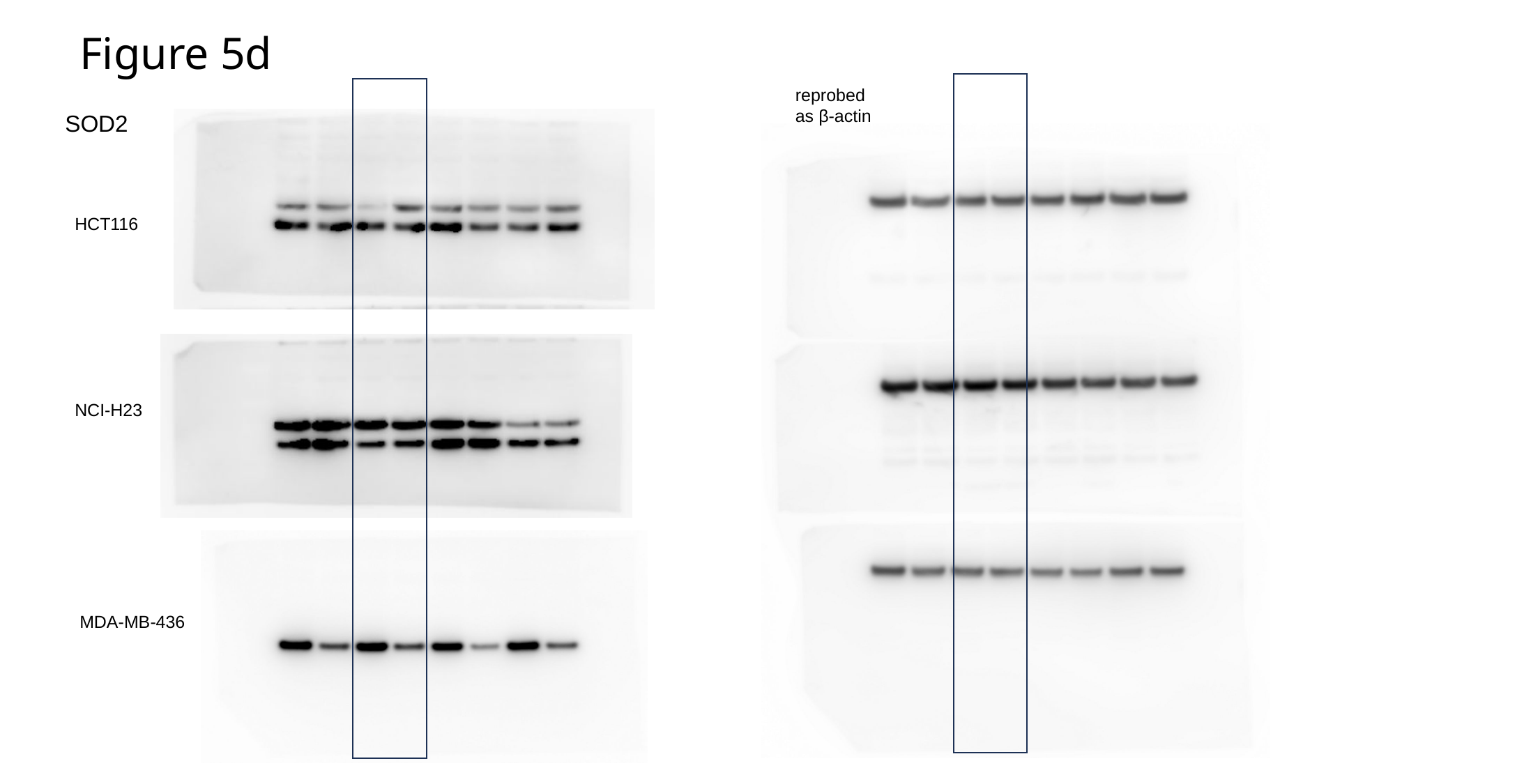

# Figure 5d
reprobed as β-actin
SOD2
HCT116
NCI-H23
MDA-MB-436

## Slide 6
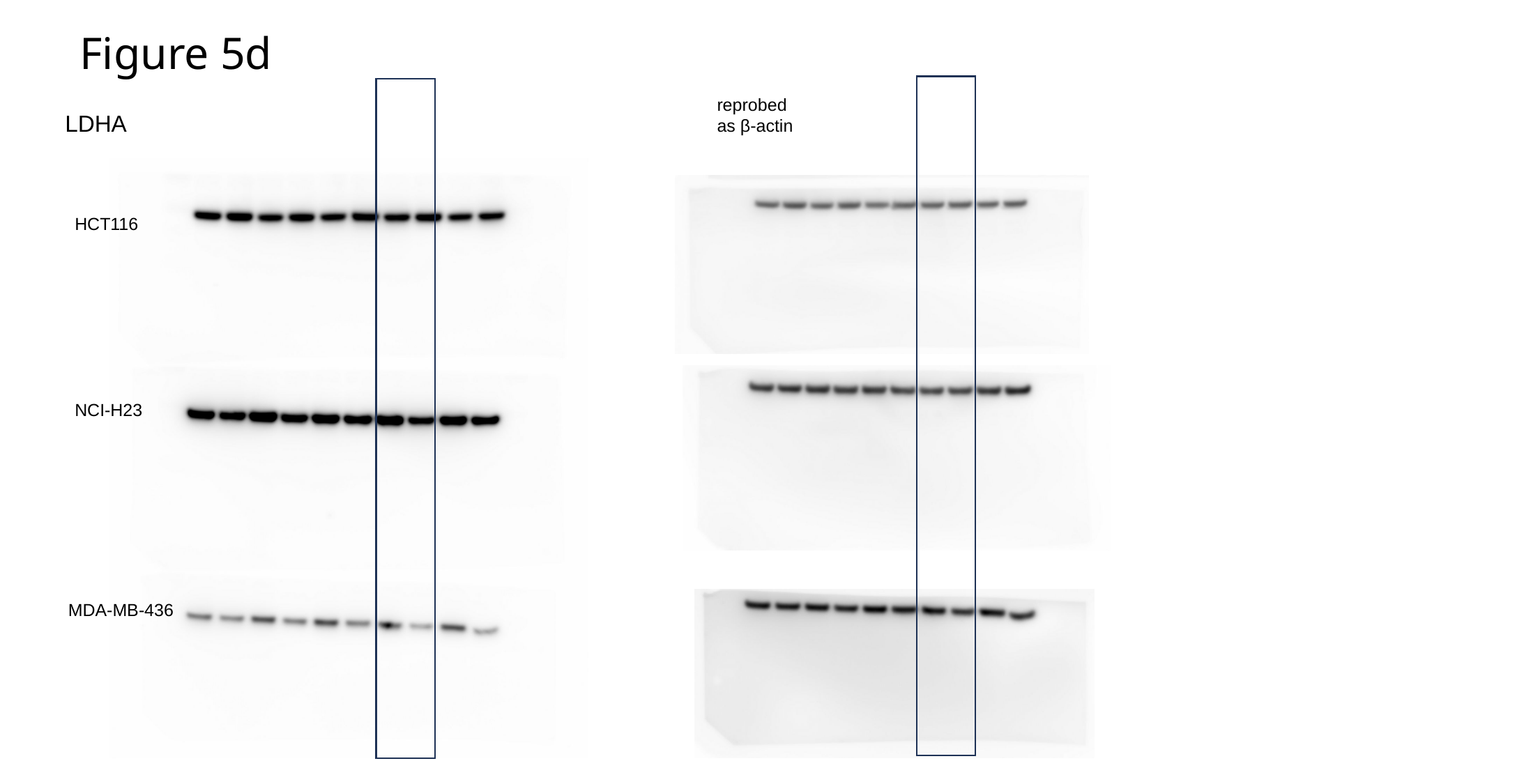

# Figure 5d
reprobed as β-actin
LDHA
HCT116
NCI-H23
MDA-MB-436
